# Tuning the Solubility of Self-Assembled Fluorescent Aromatic Cages Using Functionalized Amino Acid Building Blocks

**DOI:** 10.3389/fchem.2019.00503

**Published:** 2019-07-16

**Authors:** Marcin Konopka, Piotr Cecot, Sébastien Ulrich, Artur R. Stefankiewicz

**Affiliations:** ^1^Faculty of Chemistry, Adam Mickiewicz University, Poznań, Poland; ^2^Center for Advanced Technologies, Adam Mickiewicz University, Poznań, Poland; ^3^Institut des Biomolécules Max Mousseron (IBMM), UMR 5247, CNRS, Université de Montpellier, ENSCM, Ecole Nationale Supérieure de Chimie de Montpellier, Montpellier, France

**Keywords:** self-assembly, molecular cages, disulfides, fluorescence, dynamic covalent chemistry

## Abstract

We previously reported novel fluorescent aromatic cages that are self-produced using a set of orthogonal dynamic covalent reactions, operating simultaneously in one-pot, to assemble up to 10 components through 12 reactions into a single cage-type structure. We now introduce N-functionalized amino acids as new building blocks that enable tuning the solubility and analysis of the resulting cages. A convenient divergent synthetic approach was developed to tether different side chains on the N-terminal of a cysteine-derived building block. Our studies show that this chemical functionalization does not prevent the subsequent self-assembly and effective formation of desired cages. While the originally described cages required 94% DMSO, the new ones bearing hydrophobic side chains were found soluble in organic solvents (up to 75% CHCl_3_), and those grafted with hydrophilic side chains were soluble in water (up to 75% H_2_O). Fluorescence studies confirmed that despite cage functionalization the aggregation-induced emission properties of those architectures are retained. Thus, this work significantly expands the range of solvents in which these self-assembled cage compounds can be generated, which in turn should enable new applications, possibly as fluorescent sensors.

## Introduction

Due to the unique physicochemical properties and a wide range of applications, cage-type architectures enjoy unflagging interest (Hasell and Cooper, 2016; Markiewicz et al., 2017; Beuerle and Gole, 2018; Mastalerz, 2018). Of the many types of cage systems, those based on reversible covalent bonds begin to clearly dominate, mainly due to their adaptive properties (Lehn, 2015; Briggs and Cooper, 2017; Ono and Iwasawa, 2018). So far, molecular cages based on dynamic imine bonds (and/or acyl-hydrazones) and boronic esters have been the most frequently used among chemists (Beuerle and Gole, 2018; Acharyya and Mukherjee, 2019; Kołodziejski et al., 2019). The dynamic output of imine chemistry is particularly opulent (Belowich and Stoddart, 2012; Schick et al., 2019). Supramolecular architectures based on imine and/or acyl-hydrazone bonds have already found number of applications as biomolecular recognition receptors (Nial et al., 2007; Ulrich, 2019), nanocapsules (Durot et al., 2014; Eichstaedt et al., 2019; Jedrzejewska and Szumna, 2019), sensors (Stefankiewicz and Lehn, 2009; Bravin et al., 2019), and self-healing materials (Roy et al., 2015; Chao et al., 2016; Drożdż et al., 2018a,b). The use of disulfide bonds, reversible through *inter alia* thiolate substitution, in the generation of complex supramolecular architectures has received much attention (Otto, 2012; Black et al., 2014; Sobczak et al., 2018), except those of cage topology that are still isolated cases in the literature (Sarma et al., 2007; Stefankiewicz et al., 2012; Stefankiewicz and Sanders, 2013; Naini et al., 2014). The nature of dynamic systems based on imine and disulfide bonds is thus well-known separately but multi-dynamic systems that employs them simultaneously are an enduring challenge (Sarma et al., 2007; Orrillo et al., 2019; Reuther et al., 2019).

Recently, we have reported a method for obtaining a new type of molecular cage-like system using a set of two orthogonal reversible reactions (disulfides and acyl-hydrazones) that occur simultaneously (Drozdz et al., 2017). This was the first report of multi-dynamic and multi-component cages selectively obtained in a one-pot process, and characterized in semi-aqueous media, by applying two distinct reversible covalent bonds. The reported cages were based on two simple building blocks: tetraphenylethylene-tetraaldehyde (TPE-Ald) being known for its fluorescence properties such as aggregation-induced emission (AIE) (Zhao et al., 2012; Mei et al., 2015; Feng et al., 2018), and cysteine hydrazide, a small, chiral molecule containing three different functional groups: amine, hydrazide, and thiol. A multiplicity of factors was determined as contributing to the exceptional selectivity in the formation of cage-system, of which the presence of dimethyl sulfoxide (DMSO) in the reaction mixture was found essential for the effective cage generation. This is related to the participation of this solvent in the oxidation of thiols into disulfides which is promoted even under slightly-acidic conditions, as previously reported (Tam et al., 1991; Atcher and Alfonso, 2013). Both thiol oxidation and disulfide exchange usually proceed under mild-basic conditions, whereas acyl-hydrazones require the presence of acid catalysts.

Therefore, the simultaneous formation of these two bonds, despite an obvious progress in this area, remains a challenging task and the use of DMSO as co-solvent helps in this regard. The presence of reversible covalent bonds in the cage structure is crucial as it allows generating thermodynamically stable products by self-correction of intermediate kinetic products, while maintaining dynamic features that are important for the responsiveness of those structures. The latter are extremely important when taking into account the potential use of such systems in the selective complexation of guest molecules or in the “self-healing” processes. The first generation of our doubly-dynamic cages, despite their interesting structural features, required the use of well-defined solvent mixture (H_2_O-DMSO with predominant content of the latter from 75 to 94%). This somewhat limited the scope of investigation of e.g., fluorescence properties only to this solvent system and the potential applications one can think of.

Therefore, to expand the scope of our cages, we decided to modify the structure of one of the building blocks in order to generate two doubly-dynamic and multicomponent cage systems presenting similar structural properties but distinct hydrophobic/hydrophilic character. We chose to take advantage of the reactive α-amine present on amino acids and thus chose to functionalized the cysteine hydrazide building block at its N-terminal. It was of course necessary to ensure firstly that the preferential formation of cage structure would be retained, the principal intent simply being to modify the solubility of the cages, then to characterize the spectroscopic properties of the cages in a wider range of solvents. We describe here our syntheses of cages derived from modified cysteine hydrazide units and the properties of these new architectures in various media.

## Results and Discussion

### Design of Building Blocks

**Figure 1:**
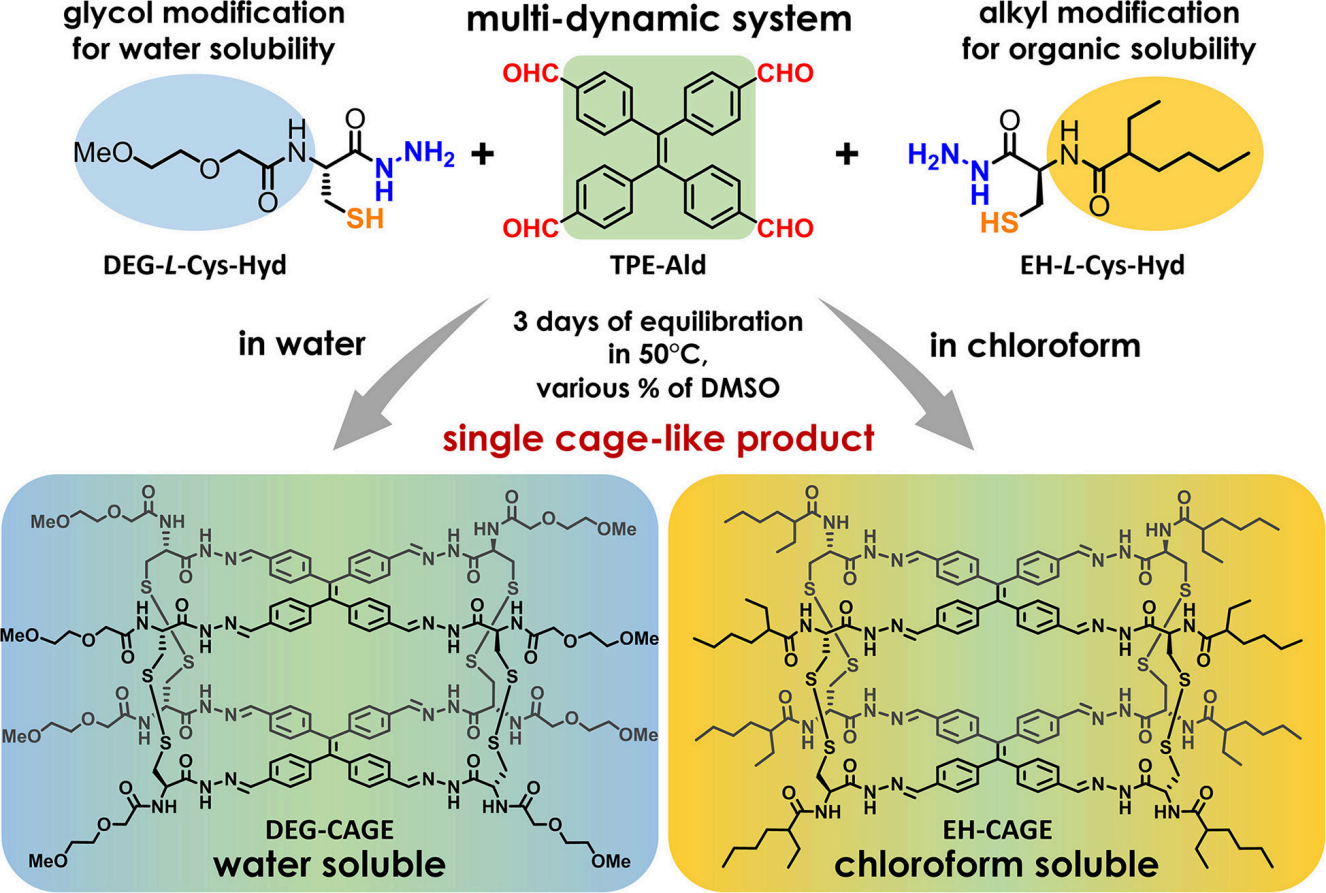
General scheme of the molecular cages formation process. Three functional groups involved in the self-assembly process were marked with colored atom labels (aldehyde: red; hydrazide: blue; thiol: orange). Structural modifications that change the physical properties of the cage have been shown in background color (light-blue for diethyl glycol moiety and light-yellow for ethyl-hexyl moiety). The color-gradient under the structure of each cage shows the contribution of individual fragments in the solubility property of the entire system.

Our investigations began with the design and selection of several structurally distinct molecular components for the efficient construction of doubly dynamic tetrapodal cage systems. As shown in Figure 1, such cages can be formed in a self-assembly process between two tetratopic components constituting upper and lower planes of the cage and four ditopic molecules as bridging linkers. Both types of building blocks must have functional groups that allow facile formation of reversible covalent bonds such as acyl-hydrazones and disulfides. The TPE aldehyde (TPE-Ald) was retained as the aromatic core of the cage-like structures, because we wanted our new cages to have an analogous structure to those reported previously (Drozdz et al., 2017). We decided to use the amino group in cysteine hydrazide to insert a structural extension *via* amide bond formation. Coupling reactions based on different amino acid systems are well-described and run under mild conditions. We decided to use two organic acids to modify the cysteine hydrazide. We chose a more polar and hydrophilic component, 2-(2-methoxyethoxy)acetic acid (DEG) containing a short di-glycol fragment, in order to achieve enhanced aqueous solubility, and a non-polar group, 2-ethylhexanoic acid (EH), to increase hydrophobicity and generate cages soluble in organic solvents. It should be mentioned, that we used the racemic ethyl hexanoic acid to make the synthesis more cost effective and facile. We assumed here that one of the key features for this project is to maintain the R-configuration on the cysteine hydrazide moieties, and that the using of racemic EH acid will not affect the self-assembly process of cage formation.

### Synthesis of Building Blocks

**Figure 2:**
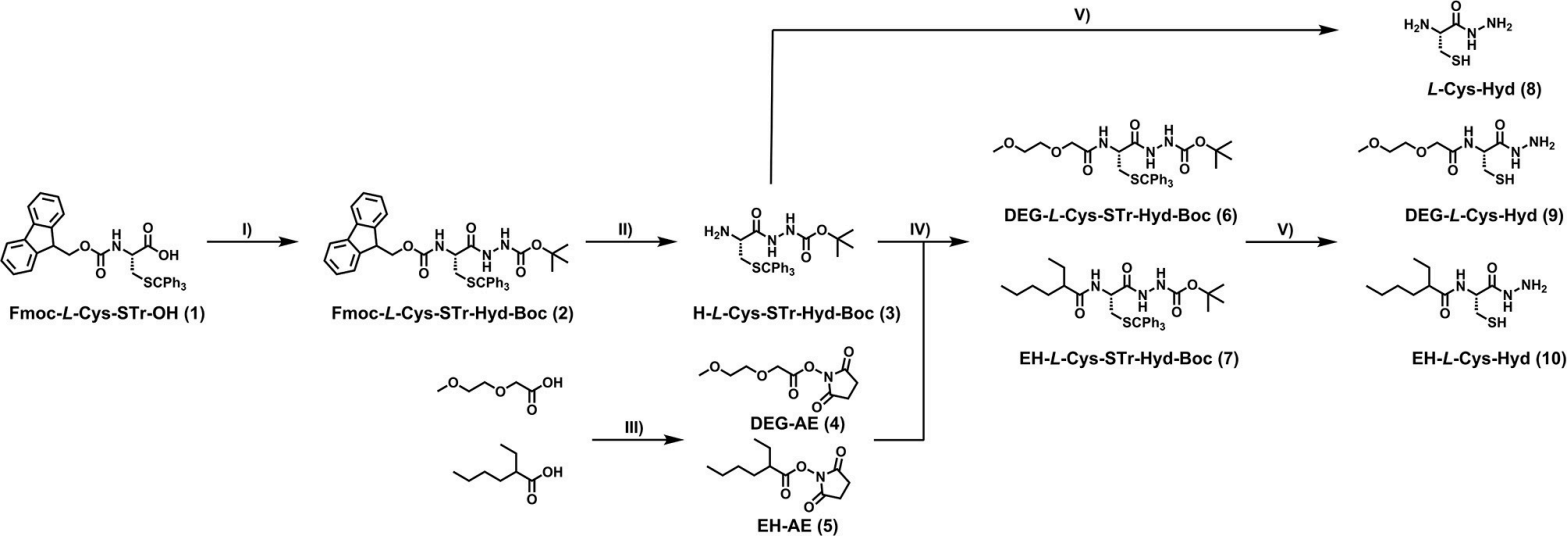
Synthesis of modified cysteine hydrazides. Reagents and conditions: (I) *tert*-butyl carbazate, EDC·HCl, HOBt, Et_3_N, DCM, 0°C to rt; (II) DMF/piperidine (8:2 v.v.), rt; (III) DCC, NHS, THF or DCM, r.t; (IV) Et_3_N, DMF, r.t.; (V) TIS/TFA (90:10 v.v.). See experimental section for details.

The syntheses (Figure 2) began with the preparation of a partially-protected cysteine hydrazide. Commercially available Fmoc-STr-*L*-Cys-OH (**1**), was coupled with tert-butyl carbazate before the Fmoc group was removed in a standard reaction with piperimidine in DMF. *N*-hydroxysuccinimide activated esters (**4** and **5**) of diethyl-glycol acid and ethyl-hexyl acid were obtained by reaction with DCC and *N*-Hydroxysuccinimide (NHS) and H-*L*-Cys-STr-Hyd-Boc (**3**) was then combined with the activated esters in amide coupling reactions. In this way, two fully protected derivatives of cysteine hydrazide tethered with DEG and EH moieties were obtained (**6** and **7**). In the final step, the hydrazide and thiol groups were deprotected in both DEG and EH derivatives in a solution of TFA/TIS 9/1. Synthetic protocols and characteristics of all obtained compounds are available in the experimental section.

### Self-Assembly of Cages

**Figure 3:**
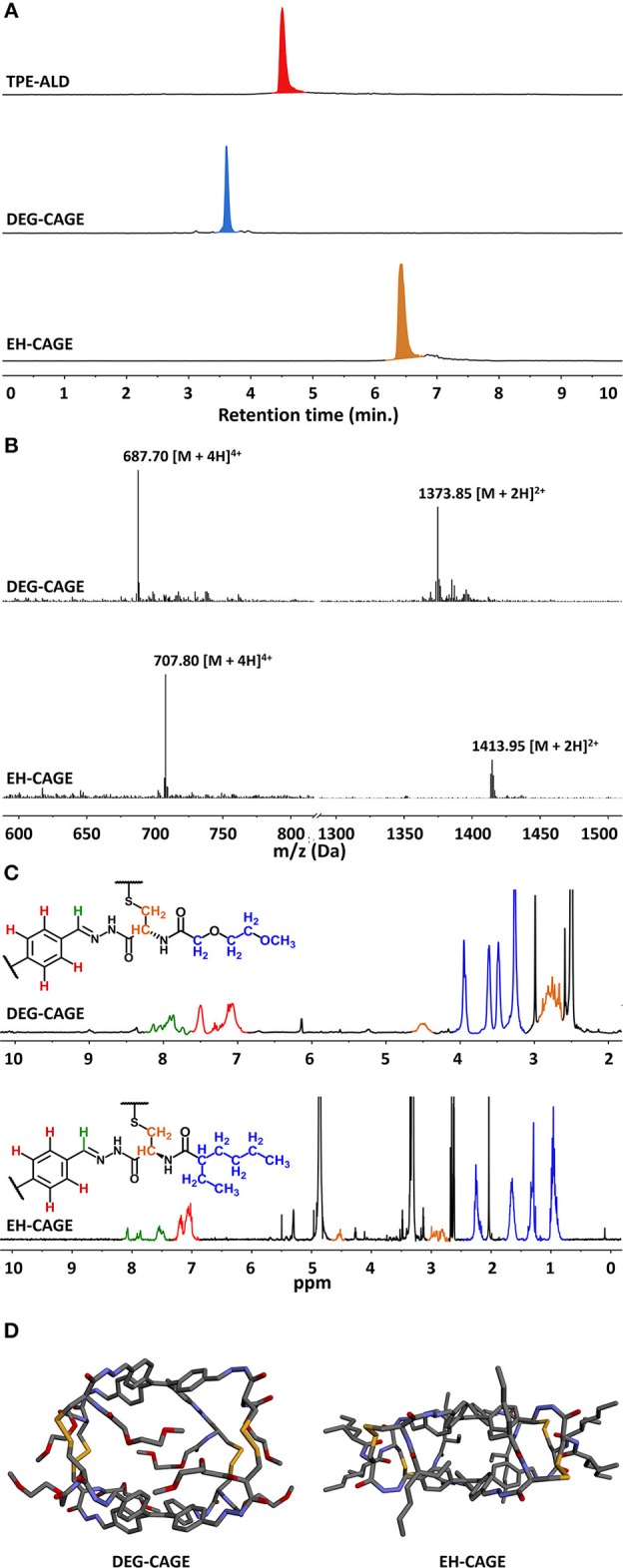
Characterization of described cages. **(A)** Comparison of HPLC chromatograms (310 nm) of TPE-ALD, DEG-CAGE and EH-CAGE showing the formation of a single product by self-assembly, **(B)** ESI-MS mass spectra of modified cages, calculated m/z for DEG-CAGE: [M+4H]^4+^ 687.2265, found 687.70, calculated m/z for [M+2H]^2+^ 1373.4458, found 1373.85; calculated m/z for EH-CAGE [M+4H]^4+^ 707.3408, found 707.80, calculated m/z for [M+2H]^2+^ 1413.6743, found 1413.95, **(C)** ^1^H NMR spectra of DEG-Cage (top) and EH-Cage (bottom). Significant peaks were marked according to the color pattern on the partial structures of the cages, **(D)** Optimized models of cages, calculated approx. sizes for DEG-Cage r = 10 Å, _sph_V = 4,200 Å^3^ (left) and for EH-Cage r = 9.15 Å, _sph_V = 3,200 Å^3^ (right).

Cage formation using the new building blocks DEG-*L*-Cys-Hyd (**9**) and EH-*L*-Cys-Hyd (**10**) was first assessed in the original solvent system (DMSO/H_2_O 94/6). In both cases, LC-MS monitoring showed that complete conversion was reached after 3 days of equilibration at 50°C, with no TPE-Ald left and the appearance of a new single peak in the mass spectra that corresponded to the expected cage compounds, respectively DEG-CAGE and EH-CAGE. Cage formation using orthogonal dynamic covalent reactions, namely acyl-hydrazone and disulfide formation, was therefore confirmed with the new *N*-functionalized cysteine-derived building blocks (Figure 3). Then we varied the nature of the solvent. First, DEG-CAGE formation was studied in a solvent system with an increasing proportion of water, from DMSO/H_2_O 94/6 to 100% H_2_O. LC-MS analysis clearly demonstrated cage formation from DMSO/H_2_O 94/6 to DMSO/H_2_O 25/75 (Table 1; Electronic Supplementary Information). In samples with less than 25% DMSO, LC-MS indicated the presence of intermediates, which corresponded to acyl-hydrazone condensation products with no sign of higher structure generated by disulfide bond formation. Similarly, EH-CAGE formation was studied in organic solvents (from 100% DMSO to 100% CHCl_3_) and LC-MS showed effective cage formation up to 90% CHCl_3_ (Table 1; Electronic Supplementary Information). Here again, a minimum of 10% DMSO seems to be essential for complete cage formation, otherwise acyl-hydrazone intermediates are formed (Electronic Supplementary Information) and do not react further. At this stage, we explain the necessity of the presence of the indicated percentages of DMSO in the reaction mixture both in terms of solubility and the facilitated oxidation of thiols to disulfides. Both cages were also characterized by ^1^H NMR. To obtain the sample the original post-self-assembly mixture was evaporated to dryness and then the residue was re-dissolved in the deuterated solvent. The recorded ^1^H NMR spectra confirmed the complex structure of the tetrapodal cages that exist as a mixture of isomers (Figure 3C). Due to the high complexity of these molecules, we employed semiempirical molecular modeling (Recife Model 1) to get further insights into the structural features of the cages (Figure 3D). Based on this we estimated the sizes and volumes of each cage. Optimization of DEG-Cage has shown the retained internal cage cavity in comparison to the unmodified cage. The lengths and shape of side chains allow them to fit fully into the side grooves of the capsule, which causes the enhancement of molecule size and volume (~4,200 Å^3^ of spherical volume). The EH-Cage side chain after optimization has no visible cavity inside the molecule. The preferred configuration of ethyl-hexanoic chains forces the TPE backbones of the EH-cage molecule to twist slightly around the central axis, while aromatic adjust and stack on each other, which constitutes the flat shape and makes the EH-cage smaller than DEG-Cage (~3,200 Å^3^ of spherical volume).

**Table 1:** Screening of cage formation in different solvent systems using *L*-Cys-Hyd (8), DEG-*L*-Cys-Hyd (9), and EH-*L*-Cys-Hyd (10).

**Entry**	**Component^a^**	**Solvents (% vol.)**			**Cage formed**
		**DMSO**	**H_**2**_O**	**CHCl_**3**_**	
1	*L*-Cys-Hyd	94	6	–	Yes
2	DEG-*L*-Cys-Hyd	94	6	–	Yes
3	DEG-*L*-Cys-Hyd	75	25	–	Yes
4	DEG-*L*-Cys-Hyd	50	50	–	Yes
5	DEG-*L*-Cys-Hyd	25	75	–	Yes
6	DEG-*L*-Cys-Hyd	10	90	–	No
7	DEG-*L*-Cys-Hyd	5	95	–	No
8	DEG-*L*-Cys-Hyd	0	100	–	No
9	EH-*L*-Cys-Hyd	100	–	0	Yes
10	EH-*L*-Cys-Hyd	75	–	25	Yes
11	EH-*L*-Cys-Hyd	50	–	50	Yes
12	EH-*L*-Cys-Hyd	25	–	75	Yes
13	EH-*L*-Cys-Hyd	10	–	90	Yes
14	EH-*L*-Cys-Hyd	5	–	95	No
15	EH-*L*-Cys-Hyd	0	–	100	No

a *Refers to the hydrazide used in reaction with TPE-Ald*.

### Fluorescence Properties

**Figure 4:**
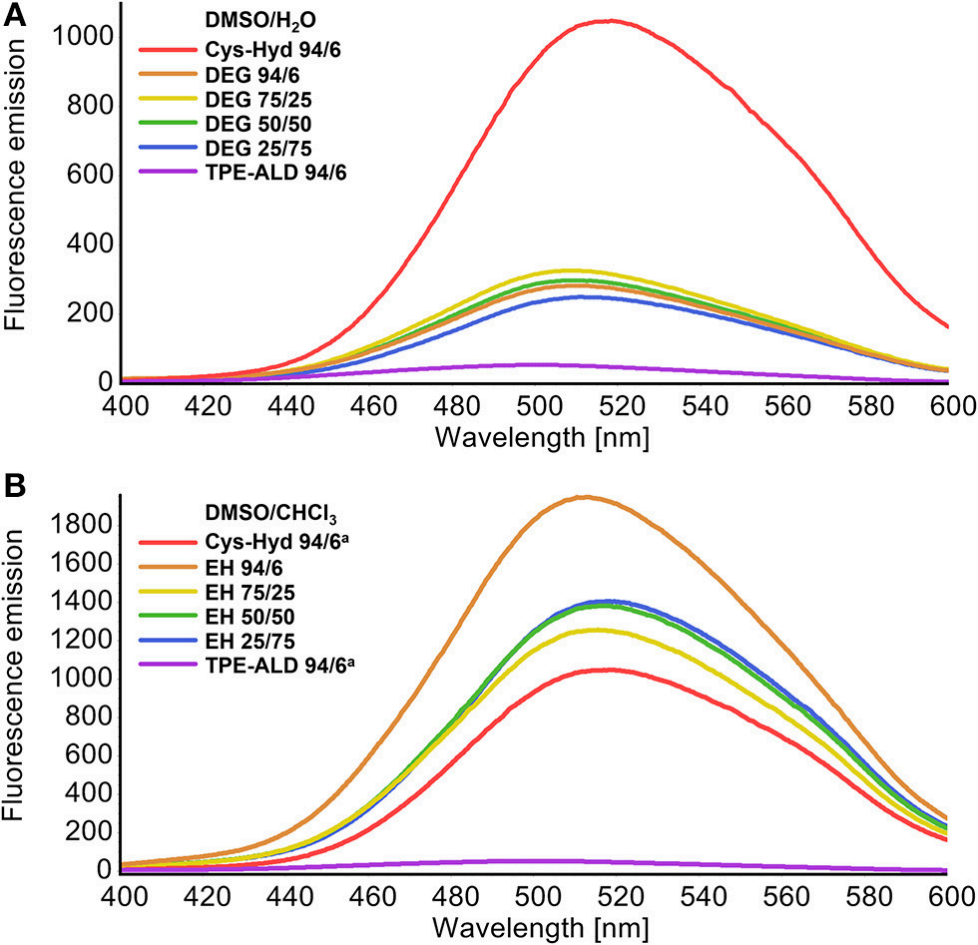
Comparison of fluorescence plots of new cages with reference to the unmodified cage (*L*-Cys-Hyd, red plot) and TPE-Ald (violet plot) (0.25 mM solutions, excitation wavelength 320 nm, range 400–600 nm). **(A)** Plots of DEG-CAGE witch various ratios of DMSO to the H_2_O. **(B)** Plots of EH-CAGE witch various ratios of DMSO to the CHCl_3_.

The fluorescence spectra of both cage compounds, DEG-CAGE and EH-CAGE, were recorded in different solvent systems at the same concentration (0.5 mM). The spectra remained essentially unaltered in all solvents, showing an emission maximum around 510–520 nm. Both cages in all solvent mixtures employed showed significant emission enhancement which can be seen by comparing with the fluorescence spectrum of TPE-ALD (Figure 4). The observed emission enhancement is caused by rigidification and suppression of phenyl rings torsion of the two TPE units within a single cage molecule (Shultz and Fox, 1989). Increasing the water content decreases about five-fold the fluorescence emission intensity of DEG-CAGE, while, on the other hand, increasing the chloroform content increases about two-fold the fluorescence emission intensity of EH-CAGE (Figure 4).

## Conclusions

We have reported herein the design and synthesis of new building blocks, based on *N*-functionalized cysteine-derived amino acids, for the one-pot multi-component self-assembly of fluorescent aromatic cages. Our results establish a convenient synthetic strategy, and show that placing such side-groups does not hinder cage formation—highlighting the robustness of the approach—and enables tuning the solubility of the corresponding cage compounds. These cages preserved interesting aggregation-induced emission properties in a now much wider range of solvent mixtures (from 90% CHCl_3_/DMSO to 75% H_2_O/DMSO), which we believe can be harnessed for sensing applications.

## Materials and Methods

Solvents and chemicals were purchased from commercial suppliers and used without further purification. Preparative purifications were performed by silica gel flash column chromatography (Merck® 40–60 μM). HPLC analyses were performed on a Waters HPLC 2695 (EC Nucleosil 300-5 C18, 125 × 3 mm column, Macherey—Nagel) equipped with a Waters 996 DAD detector. The following linear gradients of solvent A (99.9% water and 0.1% TFA) into solvent B (99.9% acetonitrile and 0.1% TFA) were used: 0–100% of solvent B in 10 min; flow 1 mL/min. Retention times are given in minutes. LC/MS analyses were performed on a Shimadzu LCMS2020 (Phenomex Kinetex C18, 2.6 μm × 7.5 cm, 100 Å) equipped with a SPD-M20A detector with the following linear gradient of solvent A (99.9% water and 0.1% HCOOH) into solvent B (99.9% acetonitrile, 0.1% HCOOH) and: 0–100% of solvent B in 10 min; flow 1 mL/min. Retention times are given in minutes. Fluorescence analyses were carried out on an AF-2500 HITACHI fluorescence spectrophotometer. UV-Vis absorption experiment was measured on UV-3100pC UVisco spectrophotometer. Samples of cages compounds were studied at 0.25 mM concentration. Excitation wavelength was set at 320 nm and emission spectra were recorded in the range 400–600 nm. ^1^H NMR, ^13^C NMR spectra were recorded at 400 MHz for ^1^H and 101 MHz for ^13^C (Bruker Avance 400) in deuterated solvents. Chemical shifts are reported in ppm relative to the solvent residual peak. HR-MS analyses were carried out at the Laboratoire de Mesures Physiques, IBMM, Université de Montpellier using Micromass Q-Tof instruments. The TPE aldehyde (TPE-Ald) and Fmoc-*L*-Cys-STr-Hyd-Boc (**2**) were obtained according to the previously reported method (Drozdz et al., 2017).

### Synthesis Protocols of Essential Chemicals and Building Blocks

#### Synthesis of H-*L*-Cys-STr-Hyd-Boc (3)

Fmoc-*L*-Cys-*S*Tr-Hyd-Boc (2.3 g, 3.31 mmol) was dissolved in a solution of DMF/piperidine (8:2, v/v; 70 mM) and then was stirred for 1 h in r.t. After that the reaction mixture was concentrated in vacuo. The crude residue was purified by flash chromatography on silica gel eluting with a gradient of DCM 100% 5 min., then 100% DCM to 90% DCM and 10% MeOH 30 min. Yield: 85%. ^**1**^**H NMR** (400 MHz, CDCl_3_) δ 7.48–7.40 (m, 6H), 7.32–7.26 (m, 6H), 7.24–7.19 (m, 3H), 3.01 (dd, *J* = 8.7, 3.9 Hz, 1H), 2.75 (dd, *J* = 13.0, 4.0 Hz, 1H), 2.57 (dd, *J* = 13.0, 8.8 Hz, 1H), 1.43 (s, 9H). ^**13**^**C NMR** (101 MHz, CDCl_3_) δ 172.00, 155.09, 144.60, 129.69, 128.13, 126.98, 81.84, 67.22, 53.48, 31.56, 28.24. **ESI-MS**: m/z calc. for [M+H]^+^ 478.2164, found 478.2161.

#### Synthesis of DEG-AE (4)

The title compound was obtained using the previously reported method for analogous compound (Meißler et al., 2016). NHS (0.8 g, 7.5 mmol) and 2-(2-methoxyethoxy)acetic acid (1.0 g, 7.5 mmol) were dissolved in 150 mL of dry THF, and then was cooled in ice bath to (0°C). DCC (1.55 g, 7.5 mmol) was added portion wise. This was stirred for 1 h at r.t. and stored in a refrigerator overnight. The DCU precipitate was filtered off and the filtrate was concentrated under vacuum to give a colorless oil. The crude product was dissolved in a small amount of THF, the resulting suspension was filtered to remove the precipitate and the procedure was repeated until a clear solution was obtained. Solvent was then removed under high vacuum to give a colorless oil. Yield: 89%. ^**1**^**H NMR** (400 MHz, CDCl_3_) δ 4.49 (s, 2H), 3.80–3.74 (m, 2H), 3.59–3.56 (m, 2H), 3.36 (s, 3H), 2.83 (s, 4H). ^**13**^**C NMR** (101 MHz, CDCl_3_) δ 168.94, 166.07, 71.89, 71.32, 66.62, 59.10, 25.66. **ESI-MS**: m/z calc. for [M+H]^+^ 232.0815, found 232.1065.

#### Synthesis of EH-AE (5)

The 2-ethylhexanoic acid (160 μL, 1.0 mmol) and *N*-hydroxycuccinimide (115 mg) were dissolved in DCM (5 mL). After 10 min. of stirring the EDC·HCl (192 mg, 1.0 mmol) was added and then the stirring was continued for 2 h at r.t. Title compound was purified by flash chromatography (gradient elution from 0 to 10% of MeOH in DCM). Yield: 72%. ^**1**^**H NMR** (400 MHz, CDCl_3_) δ 2.83 (d, *J* = 5.2 Hz, 4H), 2.58 (tt, *J* = 8.8, 5.4 Hz, 1H), 1.83–1.63 (m, 4H), 1.43–1.30 (m, 4H), 1.03 (t, *J* = 7.5 Hz, 3H), 0.91 (t, *J* = 7.1 Hz, 3H). ^**13**^**C NMR** (101 MHz, CDCl_3_) δ 171.61, 169.32, 44.92, 31.72, 29.27, 25.76, 25.64, 22.62, 13.98, 11.67. **ESI-MS**: m/z calc. for [M+H]^+^ 242.1373, found 242.1352.

#### Synthesis of DEG-*L*-Cys-STr-Hyd-Boc (6)

A solution of **DEG-AE** (231 mg, 1.0 mmol), **H-*L*-Cys-STr-Hyd-Boc** (525 mg, 1.1 mmol), and Et_3_N (0.28 mL, 2.0 mmol) in DMF (30 mL) was stirred for 24 h at room temperature. The solvent was then evaporated under vacuum. Resulted oily residue was dissolved in 1 mL of acetone and precipitated in 1 M HCl (100 mL). The white, waxy precipitate was filtered off and dried. The crude product was purified by flash chromatography on silica gel eluting with a gradient of MeOH in DCM (from 0 to 10% MeOH). The titled product was obtained as a white solid. Yield: 66%. ^**1**^**H NMR** (400 MHz, CDCl_3_) δ 7.44–7.39 (m, 6H), 7.32–7.26 (m, 6H), 7.24–7.19 (m, 3H), 4.08 (s, 1H), 4.04–3.87 (m, 2H), 3.65 (ddd, *J* = 15.8, 5.3, 3.3 Hz, 2H), 3.52 (ddd, *J* = 5.0, 3.2, 2.0 Hz, 2H), 3.32 (s, 3H), 2.78–2.58 (m, 2H), 1.42 (s, 9H). ^**13**^**C NMR** (101 MHz, CDCl_3_) δ 170.77, 169.38, 154.83, 144.44, 129.72, 128.17, 127.00, 71.60, 71.19, 70.24, 67.38, 59.06, 50.54, 32.76, 28.23. **ESI-MS**: m/z calc. for [2M+Na]^+^ 1209.5012, found 1209.5124.

#### Synthesis of EH-*L*-Cys-STr-Hyd-Boc (7)

A solution of **EH-AE** (241 mg, 1.0 mmol), **H-*L*-Cys-STr-Hyd-Boc** (525 mg, 1.1 mmol), and Et_3_N (0.28 mL, 2.0 mmol) in DMF (30 mL) was stirred for 24 h at room temperature. The solvent was then evaporated under vacuum. Resulted oily residue was dissolved in 1 mL of acetone and precipitated in 1 M HCl (100 mL). The white, waxy precipitate was filtered off and dried. The crude product was purified by flash chromatography on silica gel eluting with a gradient of MeOH in DCM (from 0 to 10% MeOH). The titled product was obtained as a white solid. Yield: 56%. ^**1**^**H NMR** (400 MHz, CDCl_3_) δ 7.44–7.40 (m, 6H), 7.26 (s, 6H), 7.23–7.18 (m, 3H), 4.18 (q, J = 7.1 Hz, 1H), 2.69 (dd, J = 13.2, 5.5 Hz, 1H), 2.59 (ddd, J = 13.1, 8.3, 2.9 Hz, 1H), 1.94–1.81 (m, 1H), 1.61–1.43 (m, 4H), 1.41 (s, 9H), 1.31–1.18 (m, 4H), 0.87–0.77 (m, 6H). ^**13**^**C NMR** (101 MHz, CDCl_3_) δ 176.83, 169.91, 154.77, 144.49, 129.64, 128.15, 126.96, 81.60, 67.24, 53.55, 49.23, 32.35, 31.05, 29.85, 28.22, 25.91, 22.87, 14.07, 12.12. **ESI-MS**: m/z calc. for [2M+Na]^+^ 1229.6092, found 1229.5532.

#### Synthesis of *L*-Cys-Hyd (8)

**H-*L*-Cys-STr-Hyd-Boc** (500 mg, 1.05 mmol) was dissolved in TFA/TIS (95/5) solution (5 mL) and stirred for 4 h at room temperature. After removal of 90% of the solvent, Et_2_O was added to the residue. The precipitate was triturated with Et_2_O and filtered. The crude material was then lyophilized twice to afford the product *L*-Cys-Hyd as a white solid. Yield: 65%. ^**1**^**H NMR** (400 MHz, CD_3_OD) δ 4.14 (m, 1H), 3.03 (qd, J = 14.6, 5.9 Hz, 2H). ^**13**^**C NMR** (101 MHz, CD_3_OD): δ = 163.32, 55.76, 25.64. **ESI-MS**: m/z calc. for [M+H]^+^ 136.0545, found 136.0541.

#### Synthesis of DEG-*L*-Cys-Hyd (9)

**DEG-*L*-Cys-STr-Hyd-Boc** (594 mg, 1.00 mmol) was dissolved in TFA/TIS (95/5 v.v.) solution (10 mL) and stirred for 4 h at room temperature. After removal of 90% of the solvent, petroleum ether was added to the residue. A white precipitate formed, which was then centrifuged and washed several times with petroleum ether. The crude material was then lyophilized twice to afford the product **DEG-*L*-Cys-Hyd** as a white solid. Yield: 78%. ^**1**^**H NMR** (400 MHz, CD_3_OD) δ 4.65–4.53 (m, 1H), 4.06 (d, *J* = 2.9 Hz, 2H), 3.74–3.70 (m, 2H), 3.62–3.58 (m, 2H), 3.41 (s, 3H), 2.93 (dq, *J* = 15.4, 7.4, 7.0 Hz, 2H). ^**13**^**C NMR** (101 MHz CD_3_OD) δ 173.02, 170.77, 72.78, 71.92, 71.08, 59.20, 55.09, 49.00, 26.49. **ESI-MS**: m/z calc. for [M+H]^+^ 252.1012, found 252.1522.

#### Synthesis of EH-*L*-Cys-Hyd (10)

**EH-*L*-Cys-*S*Tr-Hyd-Boc** (604 mg, 1.00 mmol) was dissolved in TFA/TIS (95/5 v.v.) solution (10 mL) and stirred for 6 h at room temperature. After removal of 90% of the solvent, diethyl ether was added to the oily residue. A white precipitate formed, which was then centrifuged and washed several times with diethyl ether. The crude material was then lyophilized twice to afford the product **EH-*L*-Cys-Hyd** as a white solid. Yield: 84%. ^**1**^**H NMR** (400 MHz, CD_3_OD) δ 4.47 (t, *J* = 6.9 Hz, 1H), 2.89 (dd, *J* = 14.9, 6.7 Hz, 1H), 2.80 (dd, *J* = 13.1, 7.0 Hz, 1H), 2.22 (tt, *J* = 9.6, 5.0 Hz, 1H), 1.64–1.51 (m, 2H), 1.51–1.38 (m, 2H), 1.37–1.21 (m, 4H), 0.89 (dt, *J* = 12.6, 7.0, 6.6 Hz, 6H). ^**13**^**C NMR** (101 MHz, CD_3_OD) δ 179.12, 171.09, 55.76, 39.95, 33.47, 30.74, 27.08, 23.74, 23.71, 14.32, 12.32. **ESI-MS**: m/z calc. for [M+H]^+^ 262.1573, found 262.1608.

## Data Availability

All datasets generated for this study are included in the manuscript and/or the Supplementary Files.

## Author Contributions

MK performed all organic synthesis, most experiments, analysis, and co-wrote the paper. PC performed molecular modeling and optimization of the tetrapodal cages. SU performed some analysis, interpreted the results, and co-wrote the paper. AS interpreted the results and co-wrote the paper.

### Conflict of Interest Statement

The authors declare that the research was conducted in the absence of any commercial or financial relationships that could be construed as a potential conflict of interest.

## Supplementary Material

Electronic Supplementary Information (ESI) available: NMR, MS, and HPLC chromatograms.
