# Supplementary material for: Tuning the Solubility of Self-Assembled Fluorescent Aromatic Cages Using Functionalized Amino Acid Building Blocks
Source: Front Chem. 2019 Jul 16;7:503. doi: 10.3389/fchem.2019.00503 (PMC6647868; doi:10.3389/fchem.2019.00503)
Supplement: Supplementary file 1 [file Data_Sheet_1.pdf]

Electronic Supplementary Information for:

Tuning the solubility of self-assembled fluorescent aromatic cages using functionalized amino acid building blocks.

Marcin Konopka,[1,2] Piotr Cecot,[1,2] Sebastien Ulrich,*[3] and Artur R. Stefankiewicz,*[1,2]

[1)] Faculty of Chemistry, Adam Mickiewicz University, Uniwersytetu Poznańskiego 8, 61-614 Poznań, Poland.

[2)] Center for Advanced Technologies, Adam Mickiewicz University, Uniwersytetu Poznańskiego 10, 61-614 Poznań, Poland.

[3)] Institut des Biomolécules Max Mousseron (IBMM), UMR 5247, CNRS, Université de Montpellier, ENSCM, Ecole Nationale Supérieure de Chimie de Montpellier, 8 rue de l'Ecole Normale, 34296 Montpellier cedex 5, France.

Table of contents:

1. NMR spectra of building blocks.......... 2
2. Procedure for cages formation.......... 12
3. Cages formation in different solvents.......... 12
4. Cages formation intermediates characterization.......... 14
5. NMR Spectra of cages.......... 22
6. Molecular modelling 24

1. NMR spectra of building blocks

TPE-ALD

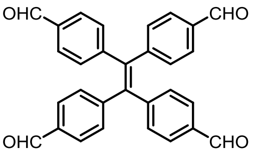

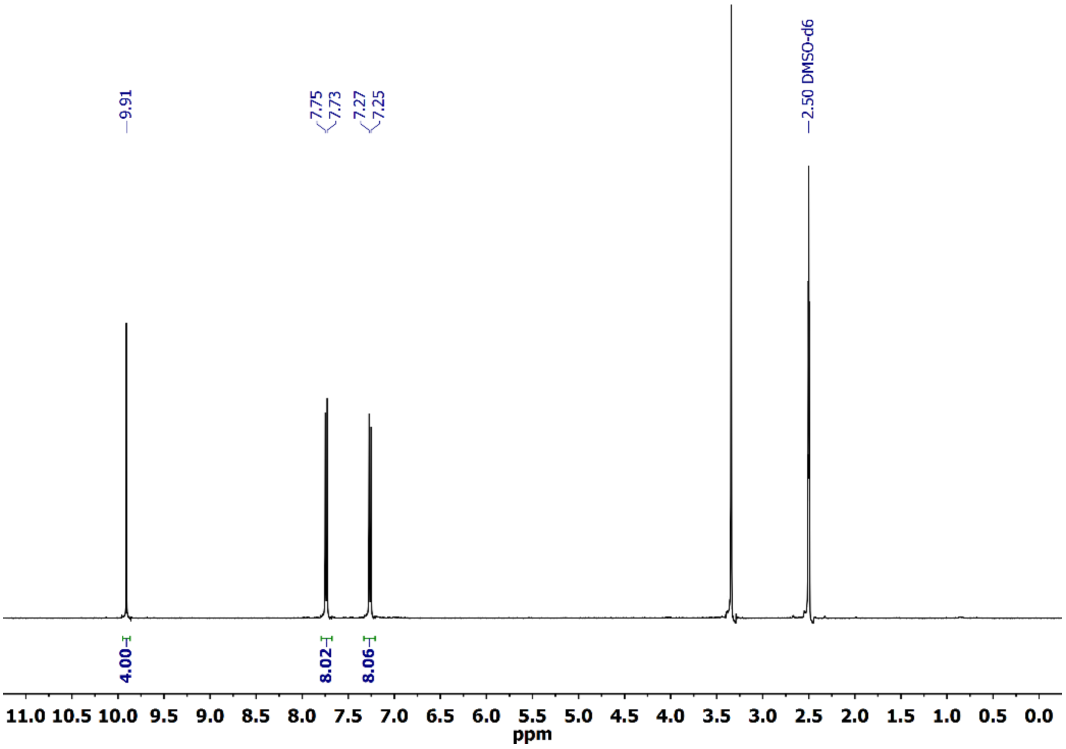

Figure S1. 1H NMR spectrum of TPE-tetra-Aldehyde in DMSO-d_6 at 298K (400 MHz).

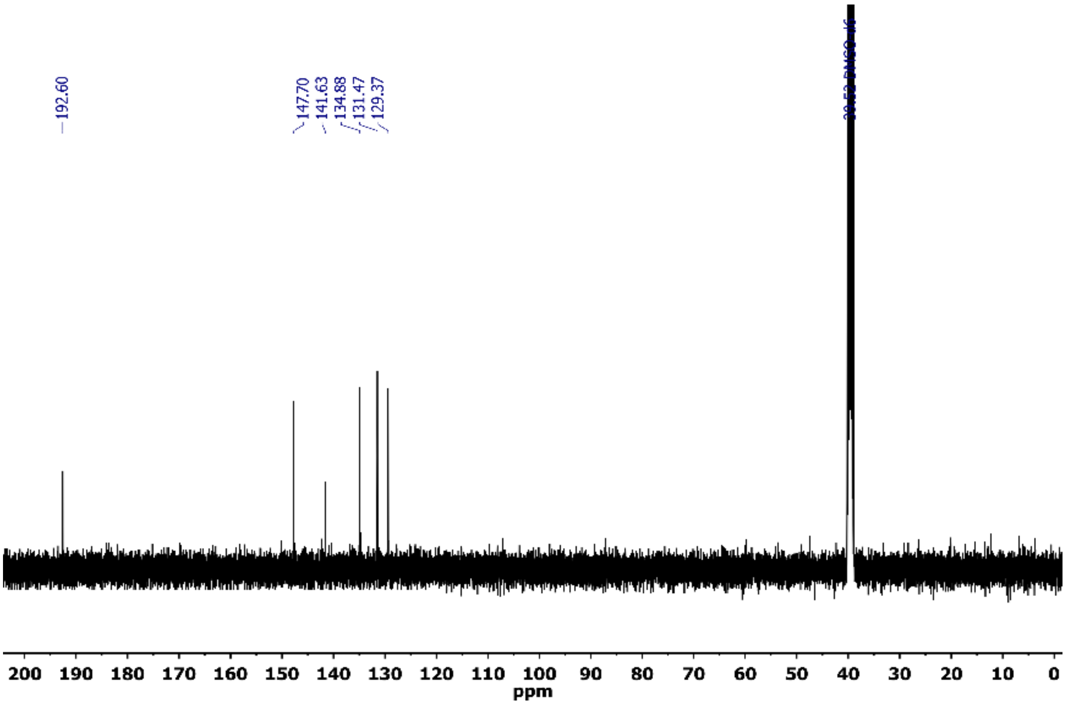

Figure S2. 13C NMR spectrum of TPE-tetra-Aldehyde in DMSO-d_6 at 298K (100 MHz).

Fmoc-*L*-Cys-STr-Hyd-Boc

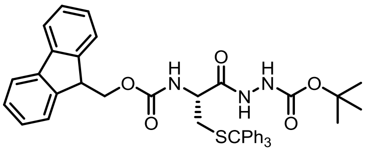

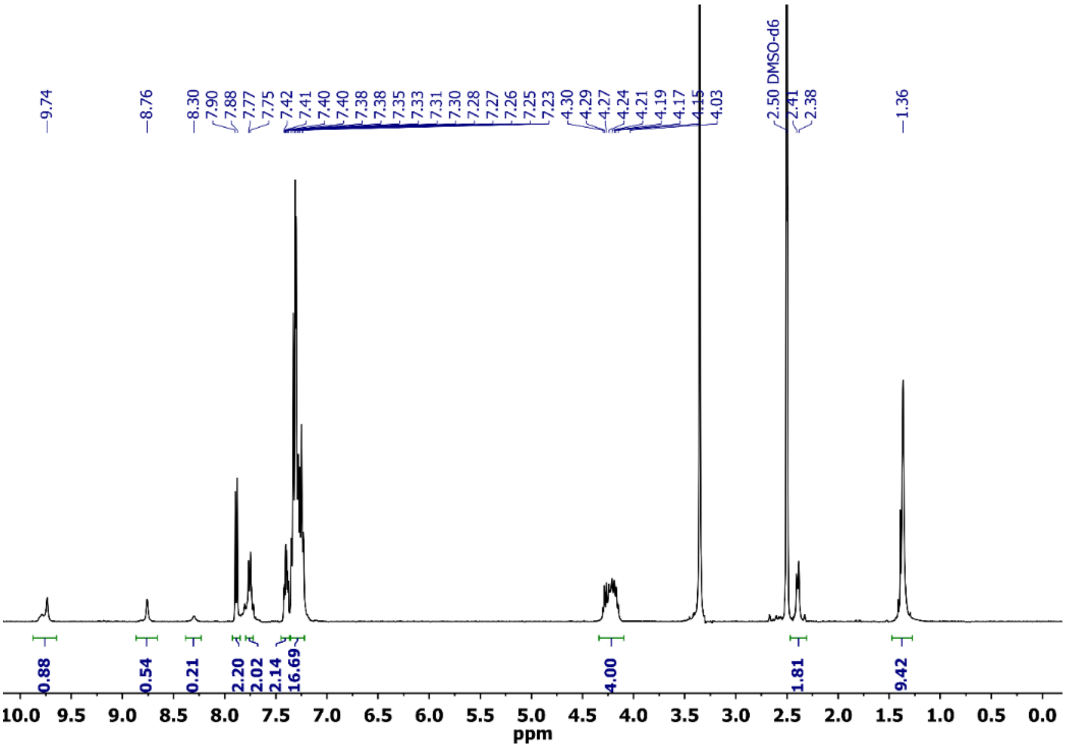

Figure S3. 1H NMR spectrum of Fmoc-*L*-Cys-STr-Hyd-Boc in DMSO-d_6 at 298K (400 MHz).

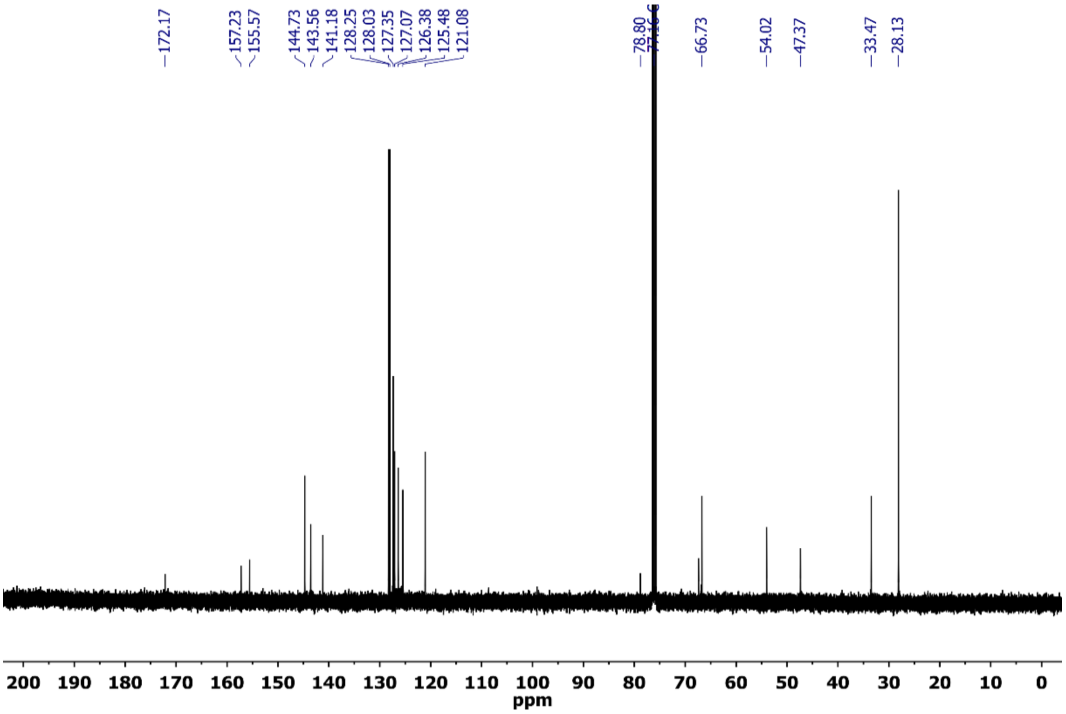

Figure S4. 13C NMR spectrum of Fmoc-*L*-Cys-STr-Hyd-Boc in DMSO-d_6 at 298K (100 MHz).

H-*L*-Cys-STr-Hyd-Boc

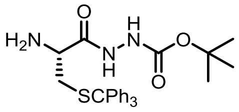

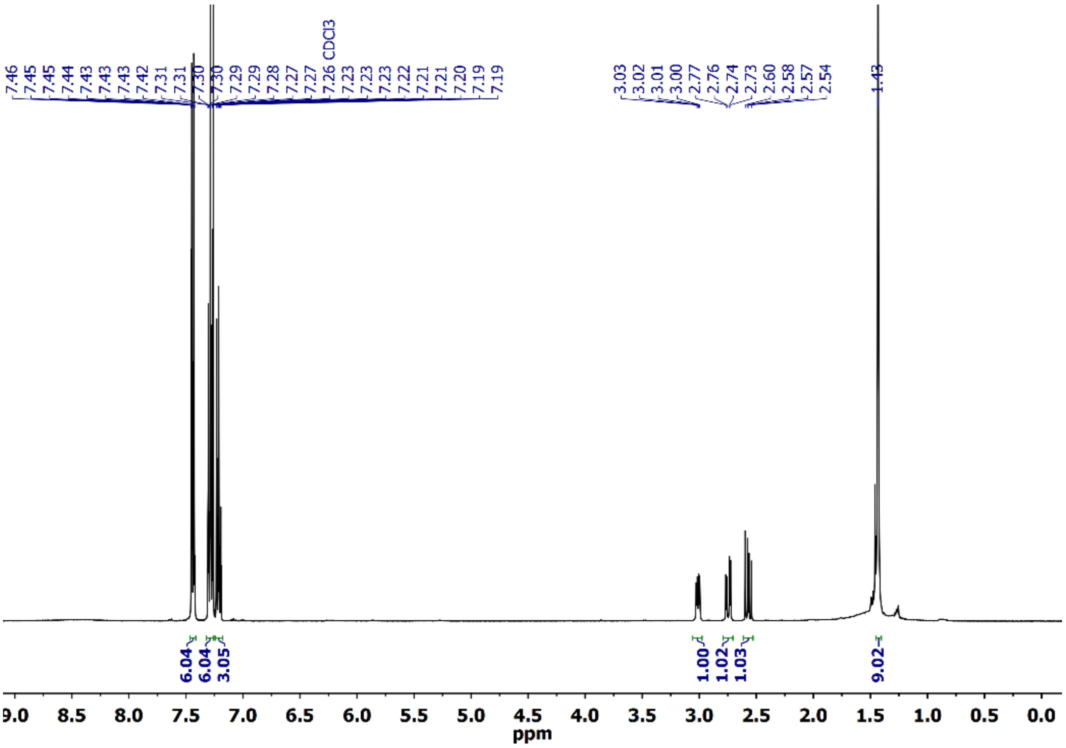

Figure S5. 1H NMR spectrum of H-*L*-Cys-STr-Hyd-Boc in $CDCl_3$-d_1 at 298K (400 MHz).

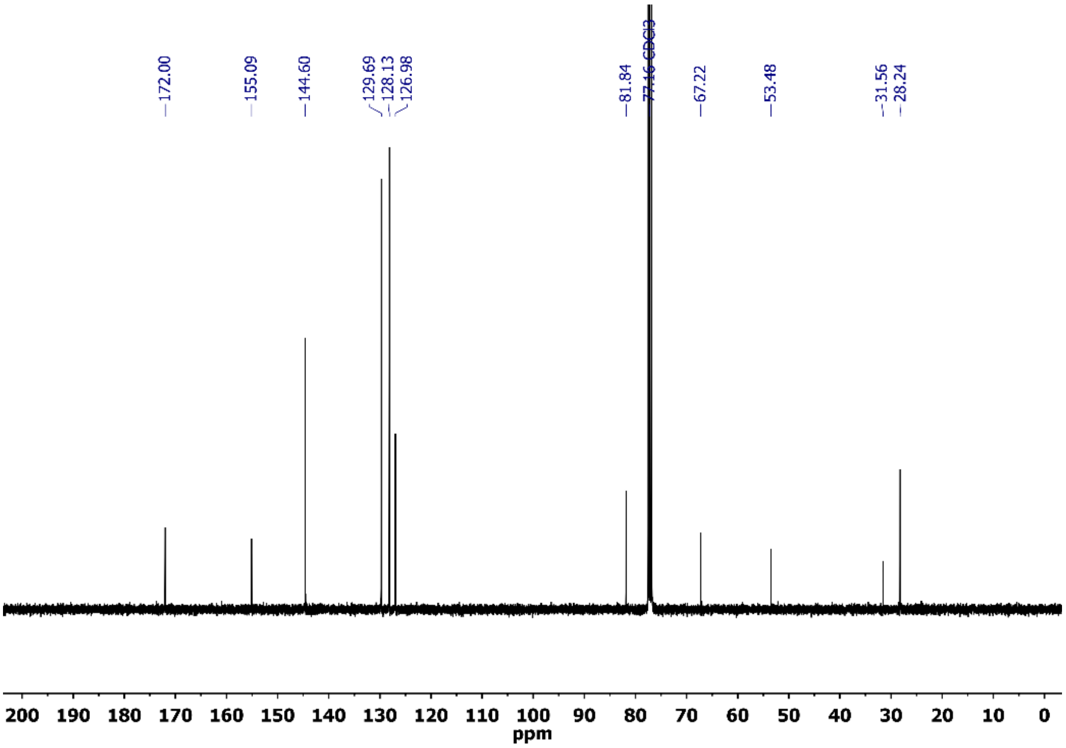

Figure S6. 13C NMR spectrum of H-*L*-Cys-STr-Hyd-Boc in $CDCl_3$-d_1 at 298K (100 MHz).

L-Cys-Hyd

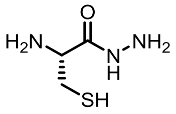

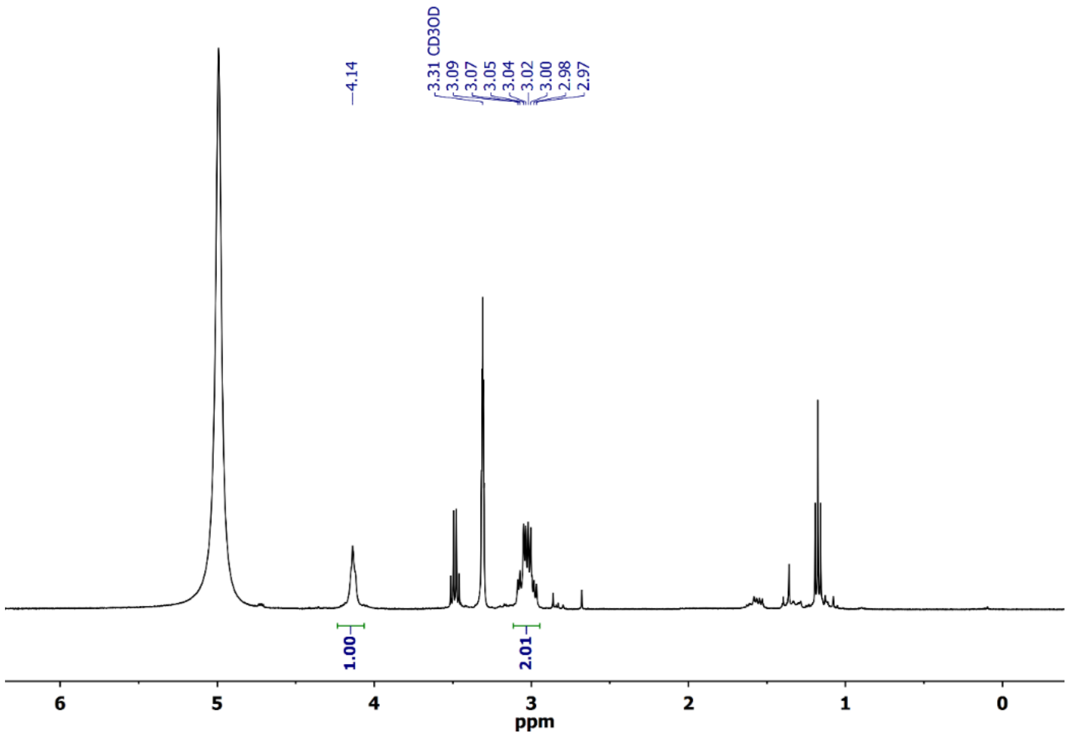

Figure S7. 1H NMR spectrum of L-Cys-Hyd in MeOD-d_4 at 298K (400 MHz).

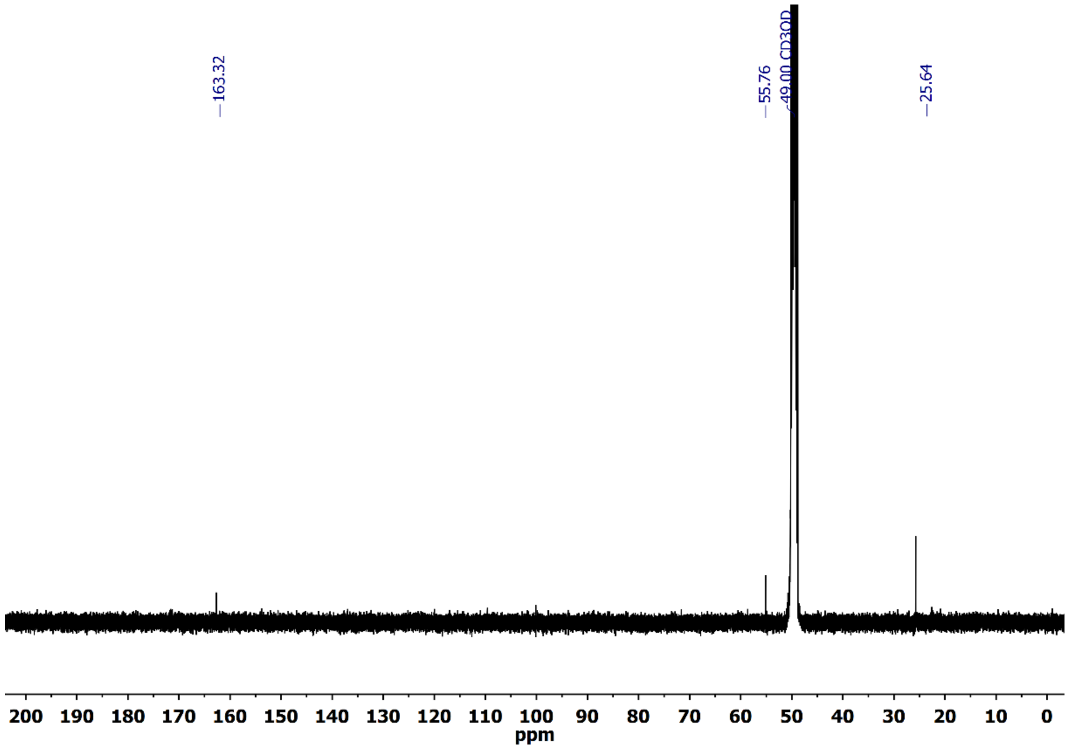

Figure S8. 13C NMR spectrum of L-Cys-Hyd in MeOD-d_4 at 298K (100 MHz).

DEG-AE

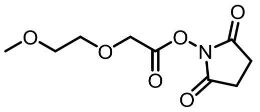

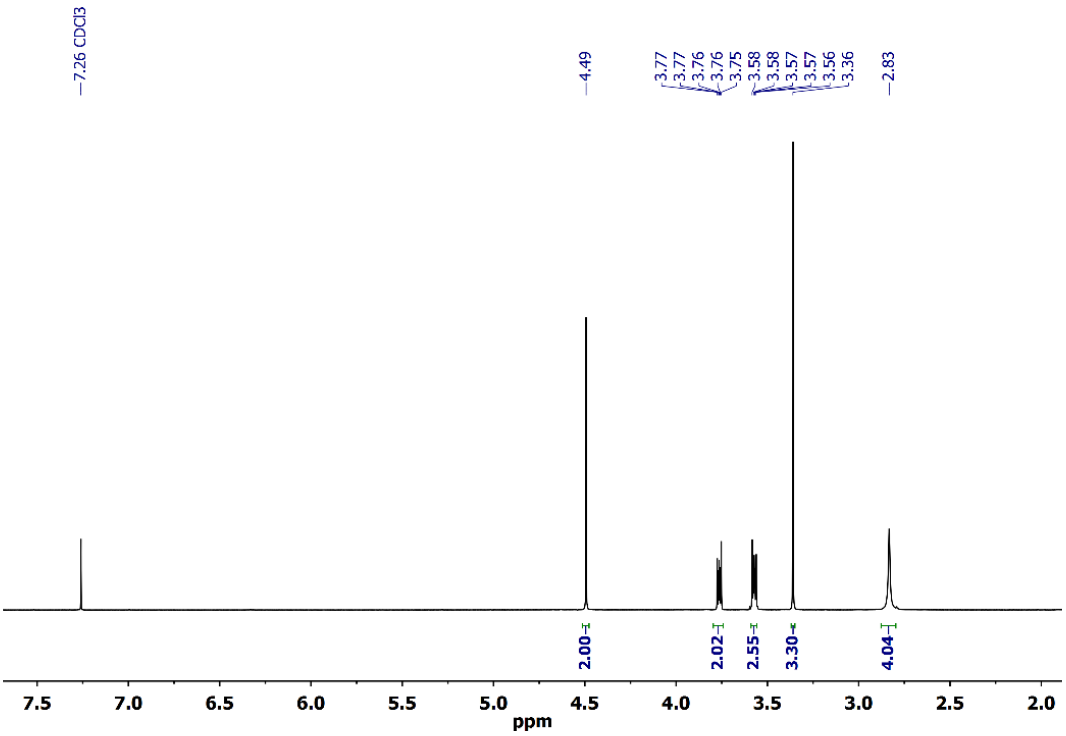

Figure S9. 1H NMR spectrum of DEG-AE in $CDCl_3$-*d_1* at 298K (400 MHz).

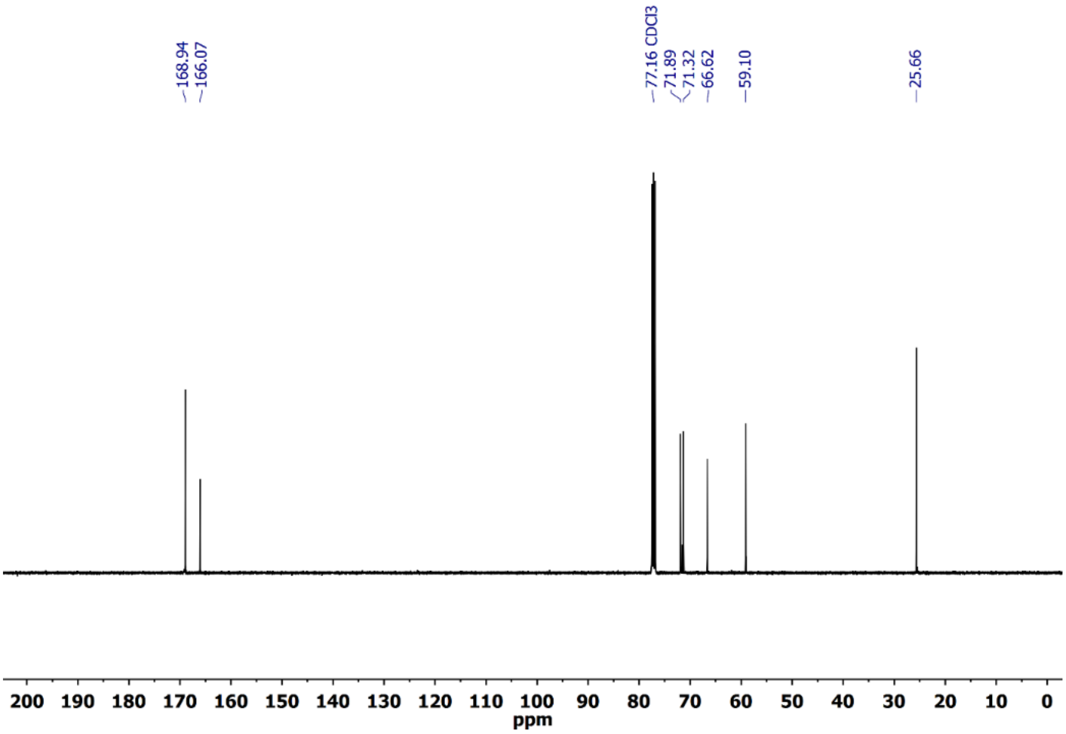

Figure S10. 13C NMR spectrum of DEG-AE in $CDCl_3$-*d_1* at 298K (100 MHz).

DEG-*L*-Cys-STr-Hyd-Boc

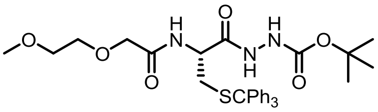

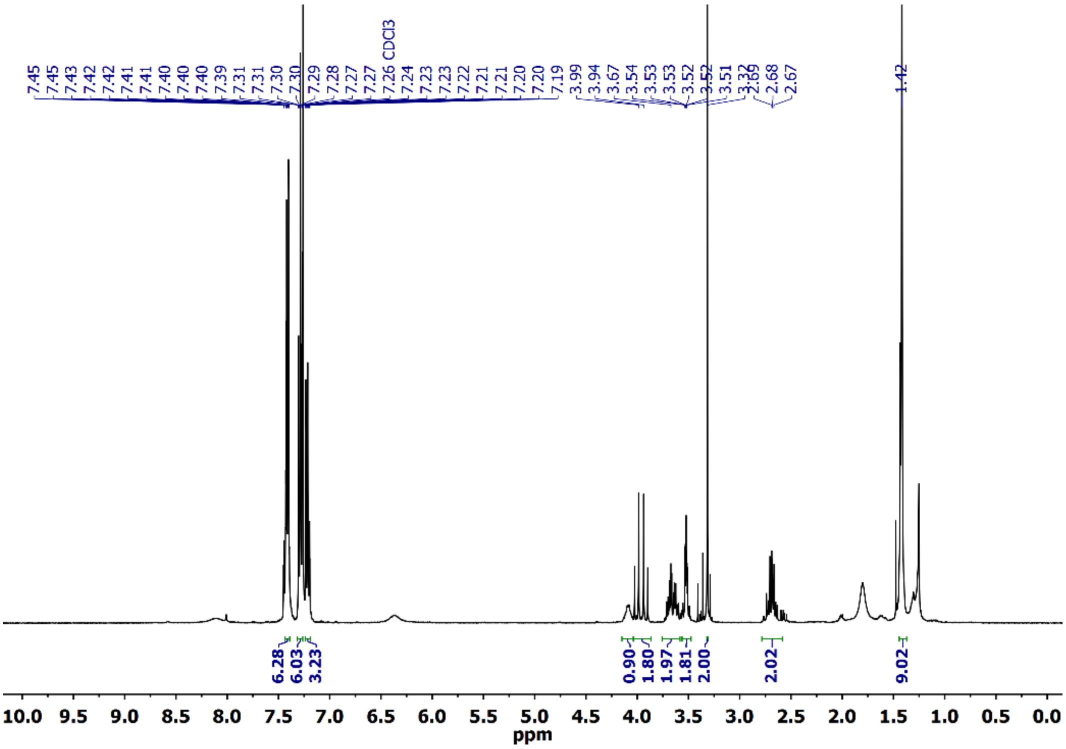

Figure S11. 1H NMR spectrum of DEG-*L*-Cys-STr-Hyd-Boc in $CDCl_3$-d_1 at 298K (400 MHz).

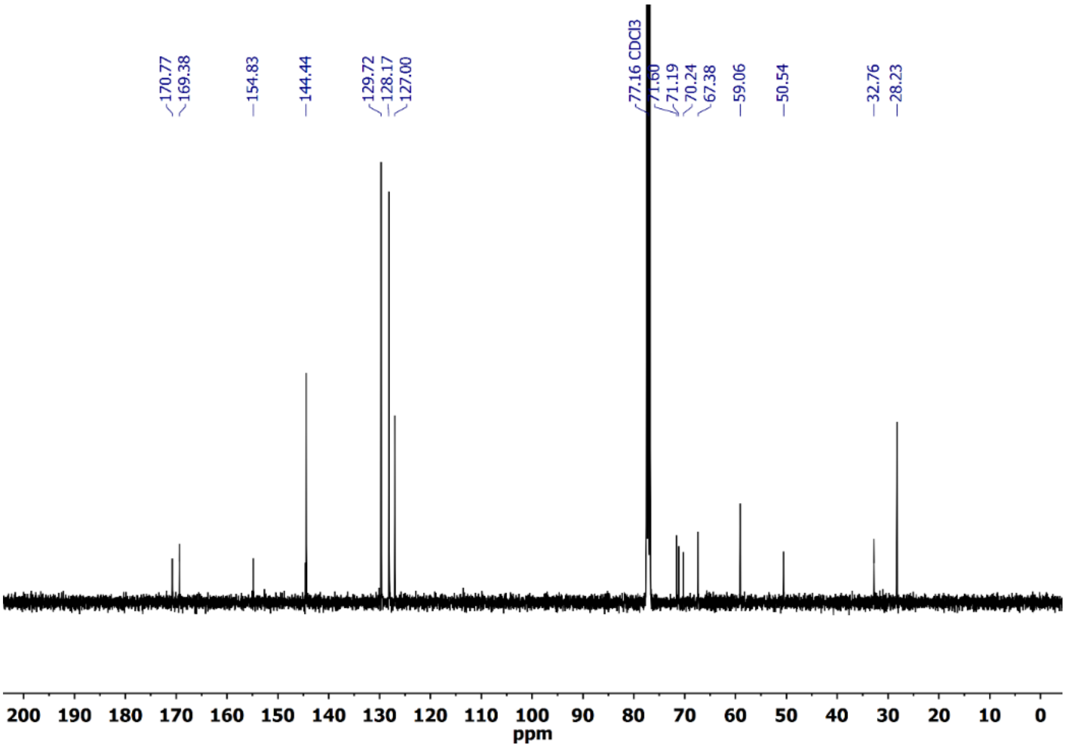

Figure S12. 13C NMR spectrum of DEG-*L*-Cys-STr-Hyd-Boc in $CDCl_3$-d_1 at 298K (100 MHz).

DEG-*L*-Cys-Hyd

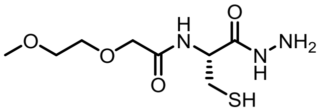

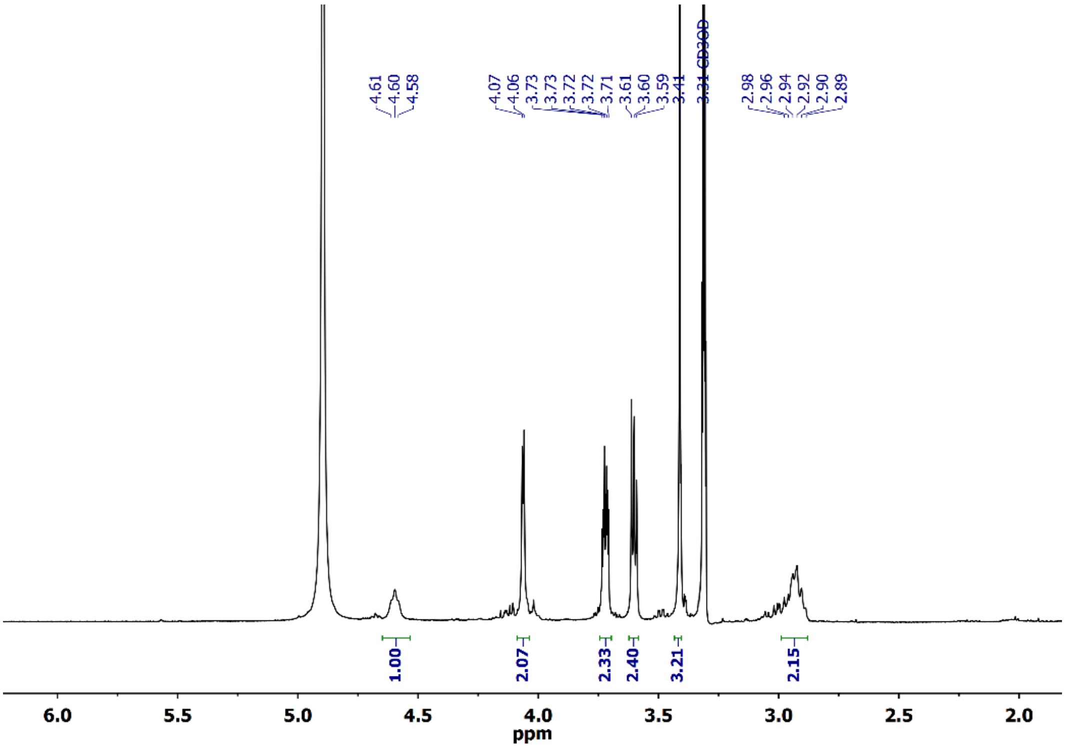

Figure S13. 1H NMR spectrum of DEG-*L*-Cys-Hyd in MeOD-d_4 at 298K (400 MHz).

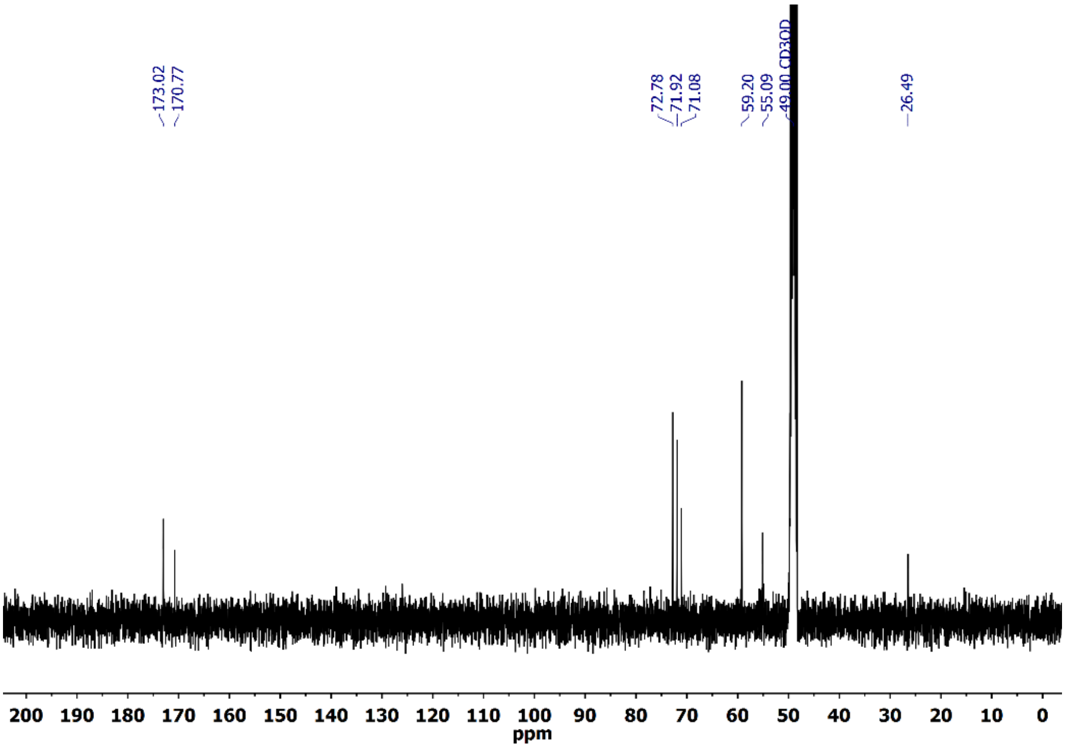

Figure S14. 13C NMR spectrum of DEG-*L*-Cys-Hyd in MeOD-d_4 at 298K (100 MHz).

EH-AE

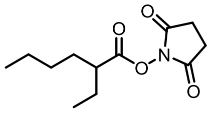

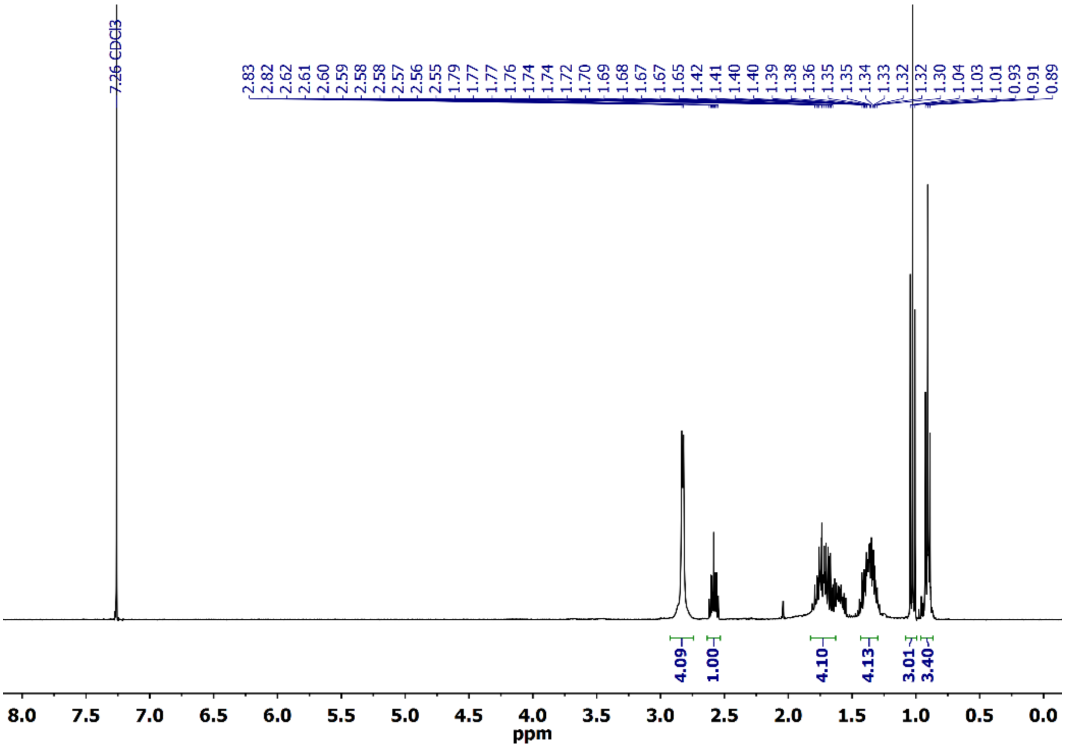

Figure S15. 1H NMR spectrum of EH-EA in $CDCl_3$-*d₁* at 298K (400 MHz).

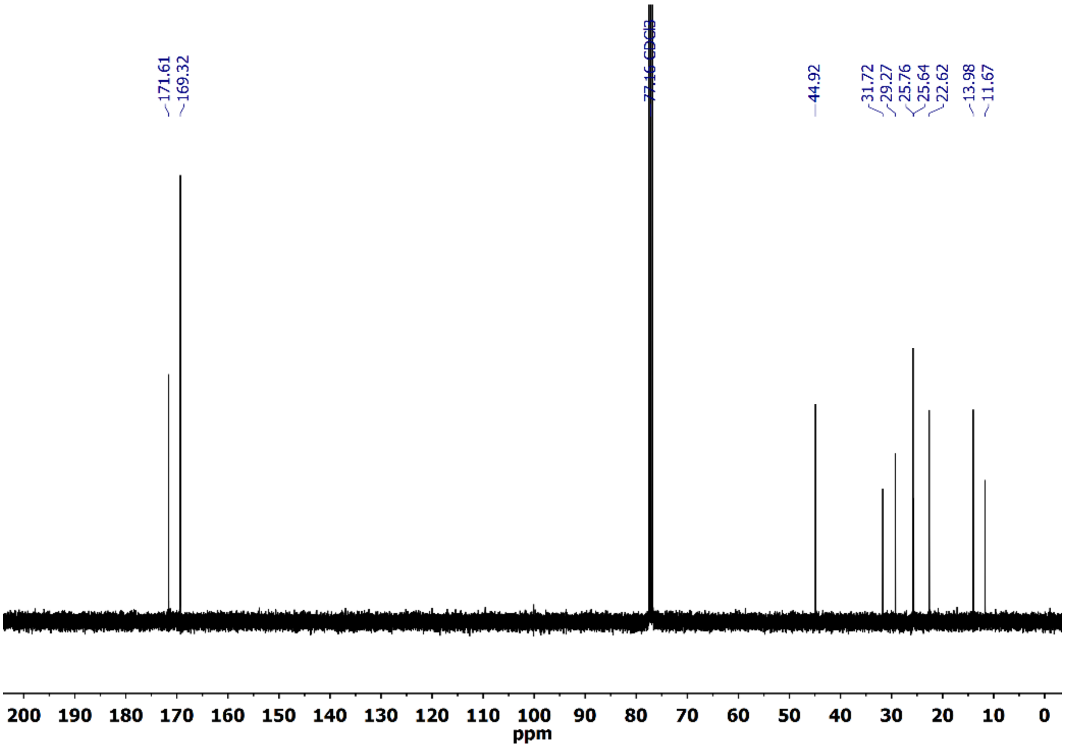

Figure S16. 13C NMR spectrum of EH-EA in $CDCl_3$-d_1 at 298K (100 MHz).

EH-*L*-Cys-STr-Hyd-Boc

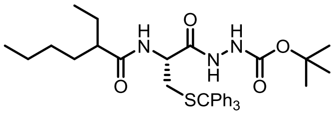

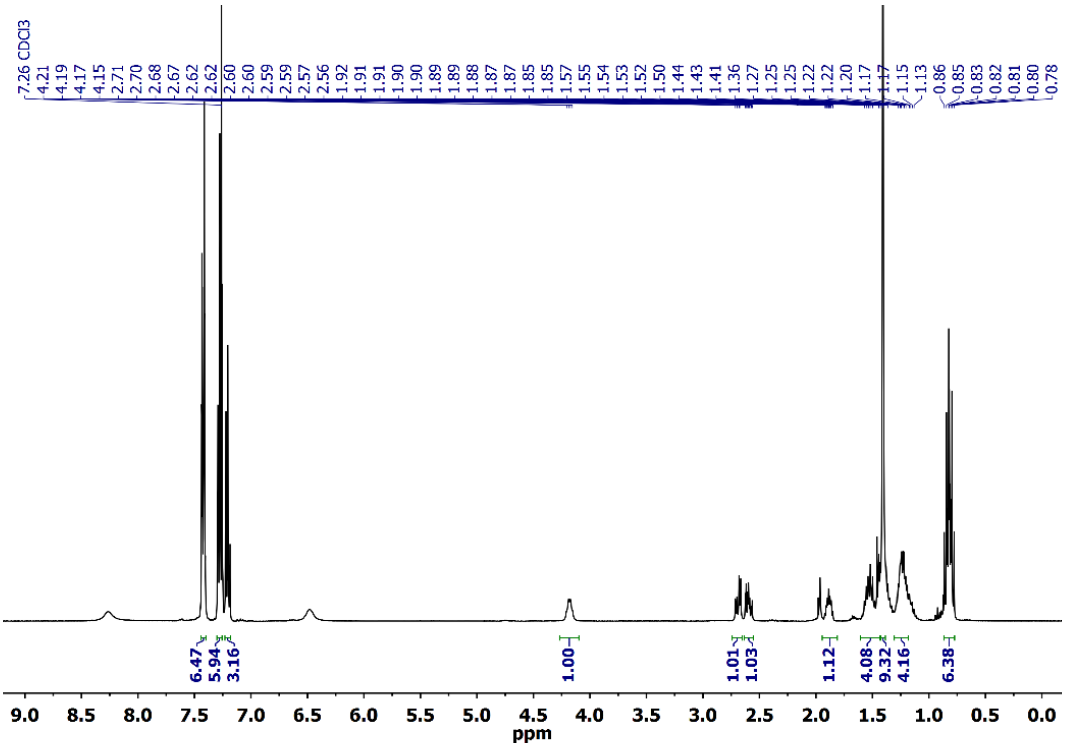

Figure S17. 1H NMR spectrum of EH-*L*-Cys-STr-Hyd-Boc in $CDCl_3$-*d_1* at 298K (400 MHz).

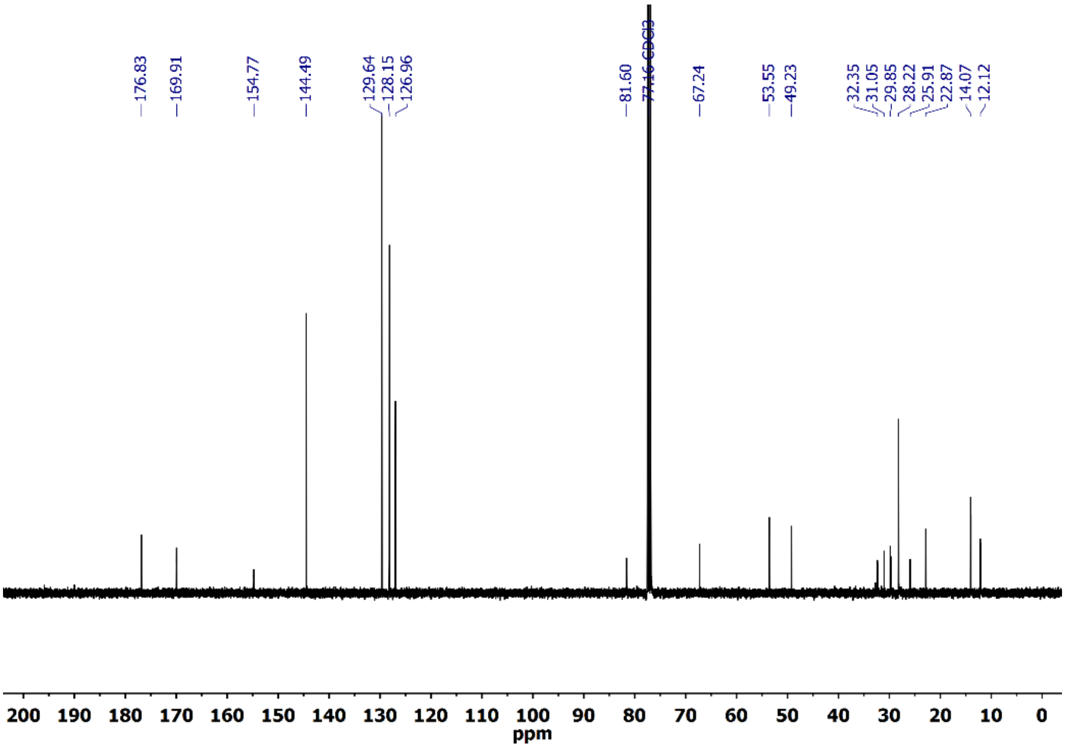

Figure S18. 13C NMR spectrum of EH-*L*-Cys-STr-Hyd-Boc in $CDCl_3$-*d_1* at 298K (100 MHz).

EH-*L*-Cys-Hyd

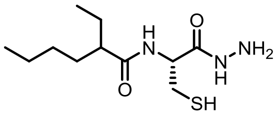

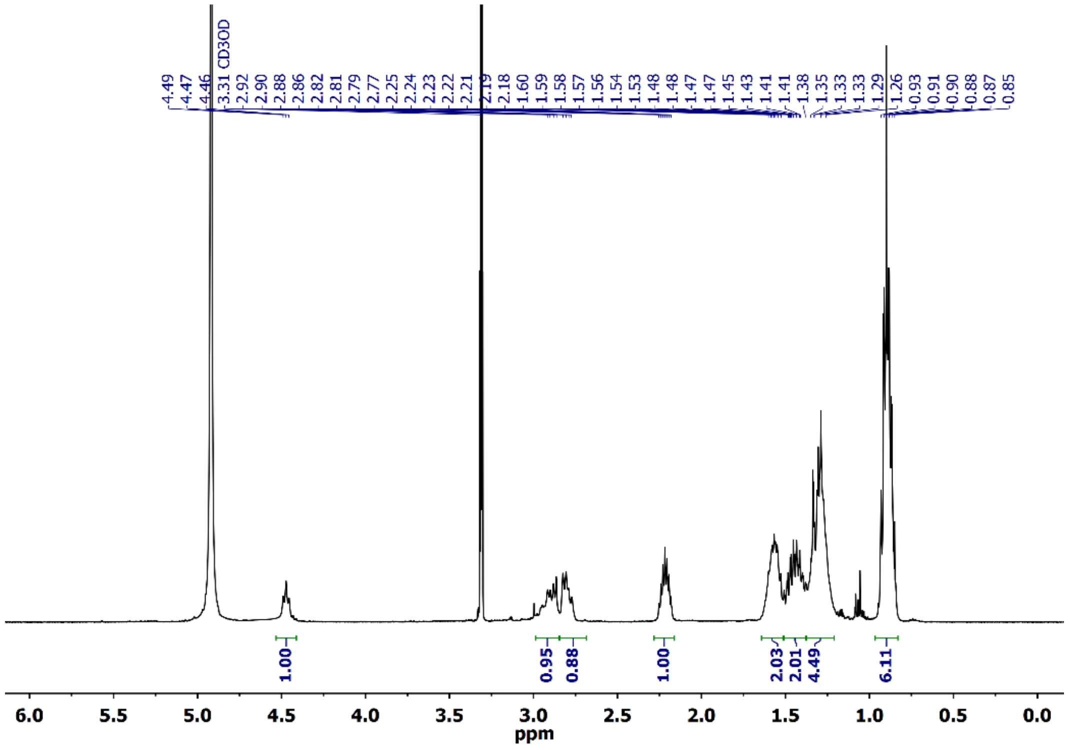

Figure S19. 1H NMR spectrum of EH-*L*-Cys-Hyd in MeOD-d_4 at 298K (400 MHz).

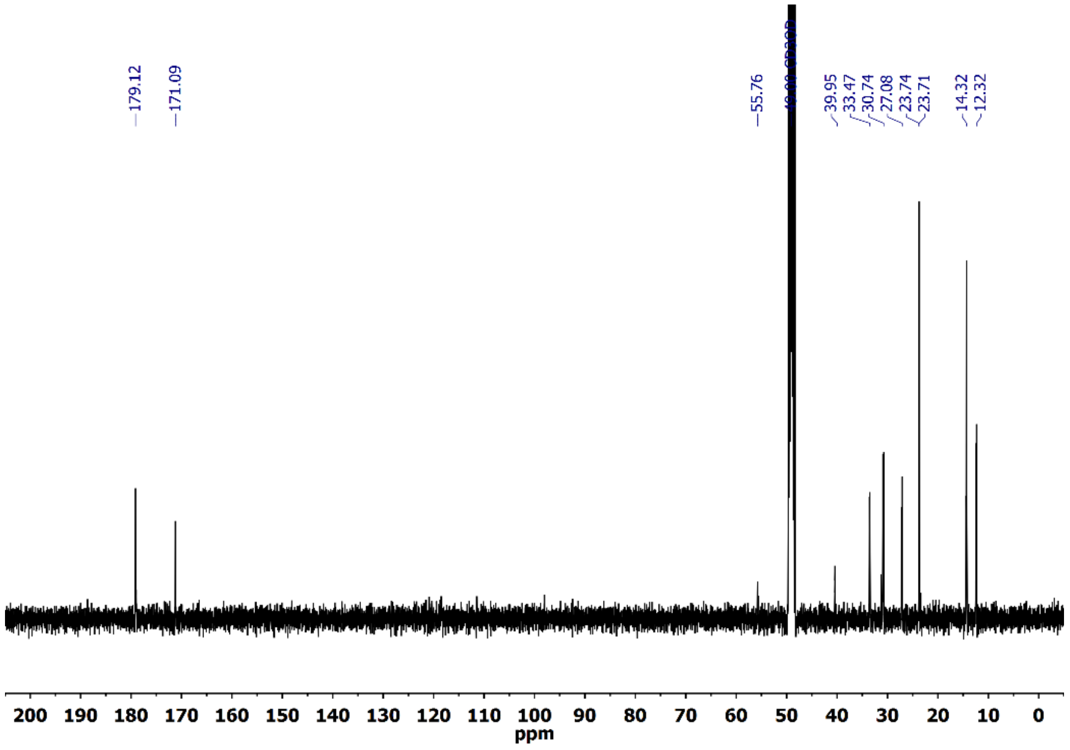

Figure S20. 13C NMR spectrum of EH-*L*-Cys-Hyd in MeOD-d_4 at 298K (100 MHz).

2. Procedure for cages formation

Stock solutions of **TPE-Ald** (50 mM) and **EH-*L*-Cys-Hyd** (150 mM) in DMSO, and **DEG-*L*-Cys-Hyd** (150 mM) in H_2O were prepared. TPE-ALD (22 mg, 0.05 mmol) was dissolved in 1.0 ml of DMSO. **EH-*L*-Cys-Hyd** (40 mg, 0.15 mmol) was dissolved in 1.0 ml of DMSO. **DEG-*L*-Cys-Hyd** (38 mg, 0.15 mmol) was dissolved in 1.0 ml of H_2O. The appropriate volume was taken to place 1.0 μmol of **TPE-Ald** and 4.0 μmol of cysteine-hydrazide derivative. Each experiment for cages formation was run in this way. Solution of **TPE-Ald** (50 mM, 20 μL) and solution of cysteine-hydrazide derivative (150 mM, 30 μL) were added into empty Eppendorf vial and then it was filled with 450 μL of solvent up to 0.5 mL of total volume. The Eppendorf vials were heated at 50 °C for 3 days and the outcome of the reaction was monitored by LC-MS.

3. Cages formation in different solvents

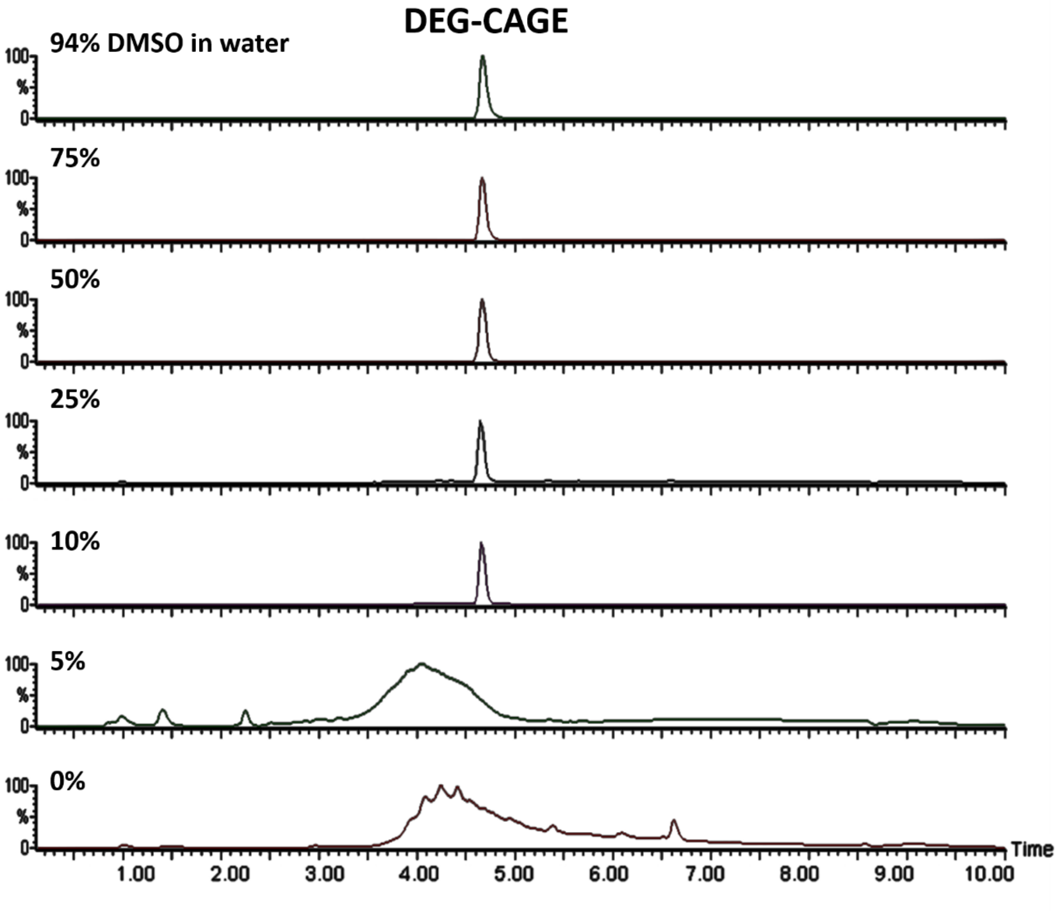

Figure S21. Comparison of HPLC chromatograms (310 nm) of **DEG-Cage** formation in various percentage of DMSO in water. System requires at least 10% of DMSO for exclusive DEG-Cage formation.

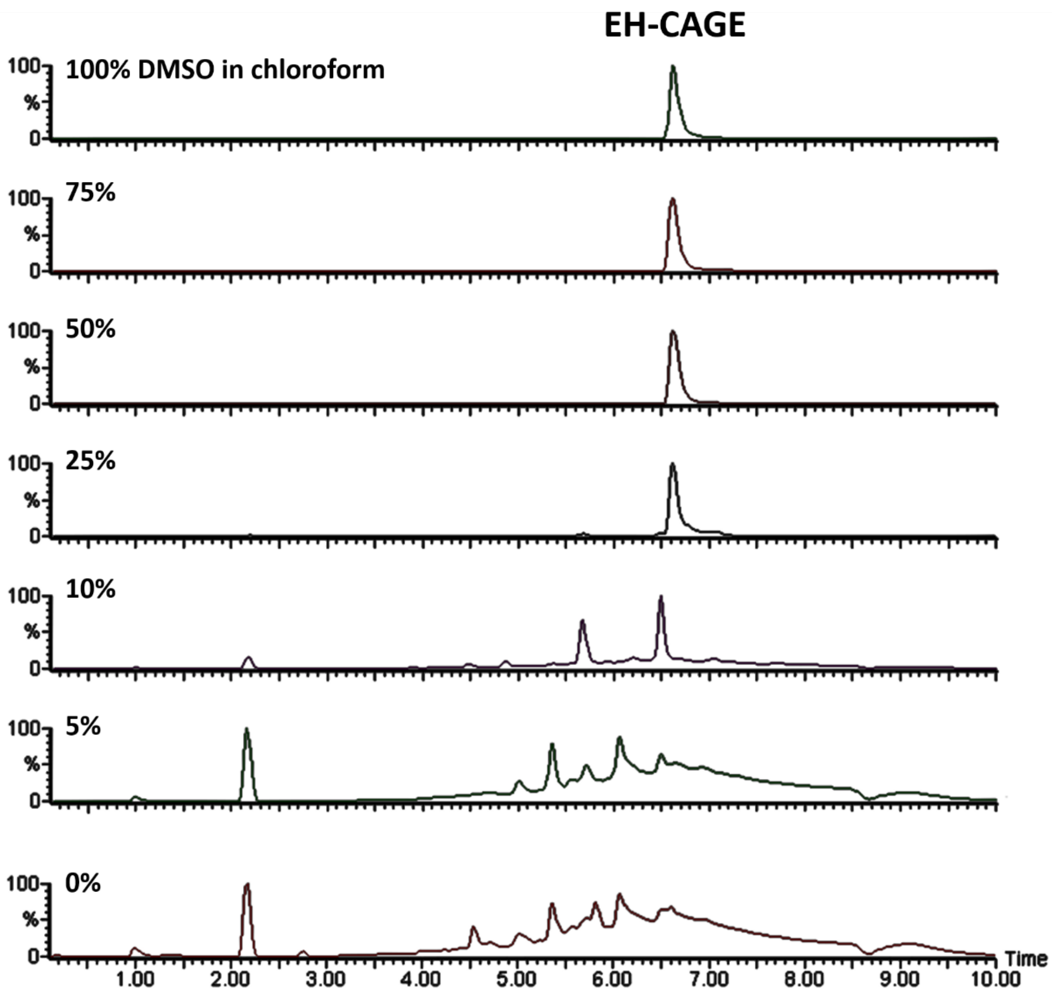

Figure S22. Comparison of HPLC chromatograms (310 nm) of **EH-Cage** formation in various percentage of DMSO in chloroform. System requires at least 25% of DMSO for exclusive **EH-Cage** formation. Partially EH-Cage formation is observed for 10% of DMSO in chloroform.

4. Cages formation intermediates characterization

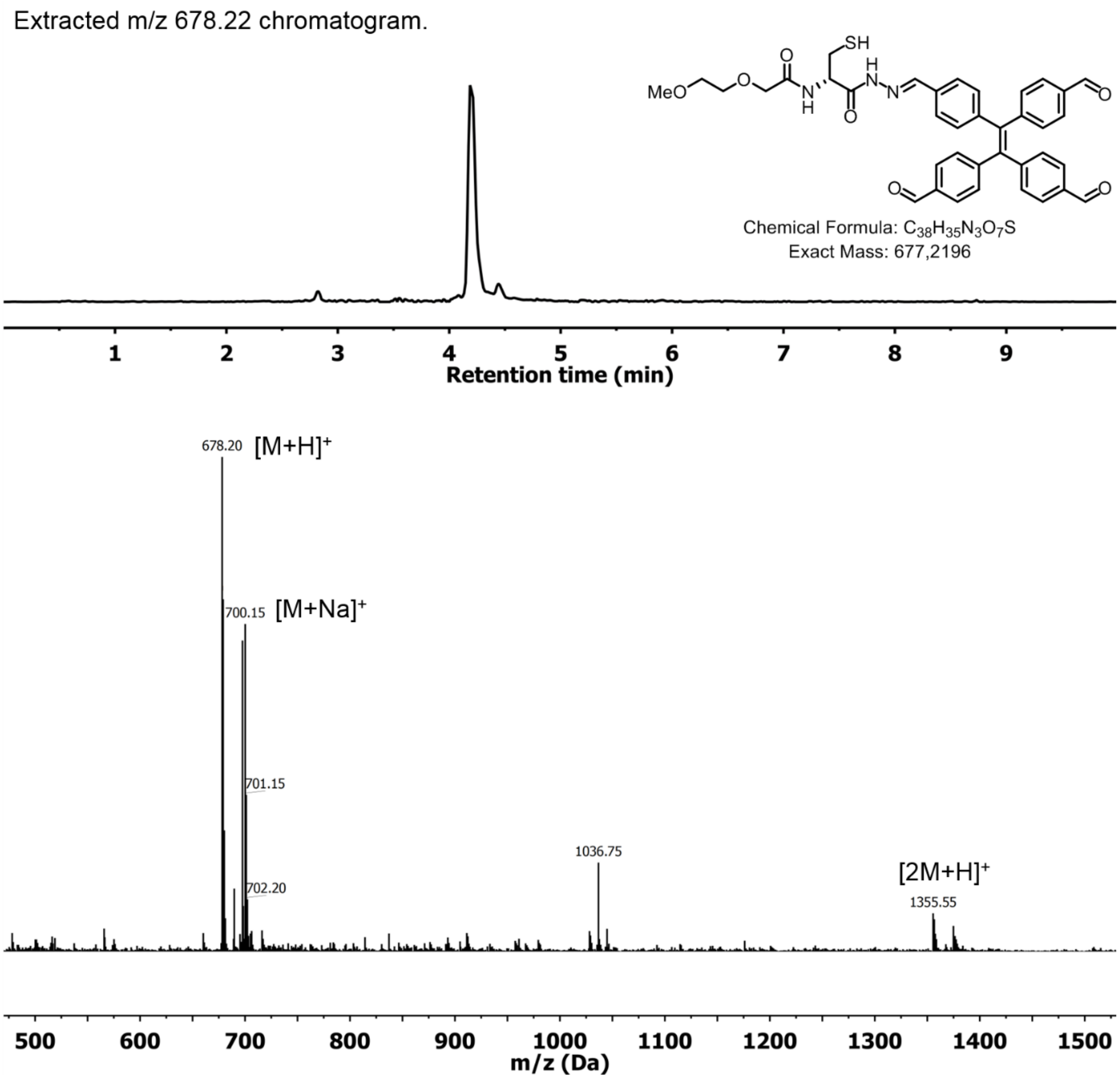

Figure S22. Extracted chromatogram for m/z 678.22 from LC-MS trace of equilibrating mixture of DEG-Cage formation after 24h (top). Mass spectra of DEG-Cage-intermediate, mono-acylhydrazone, calculated m/z for [M+H]+ 678.2269, found 678.20, calculated m/z for $[M+Na]^+$ 700.2088, found 700.15, calculated m/z for $[2M+H]^+$ 1355.4465, found 1355.55 (down).

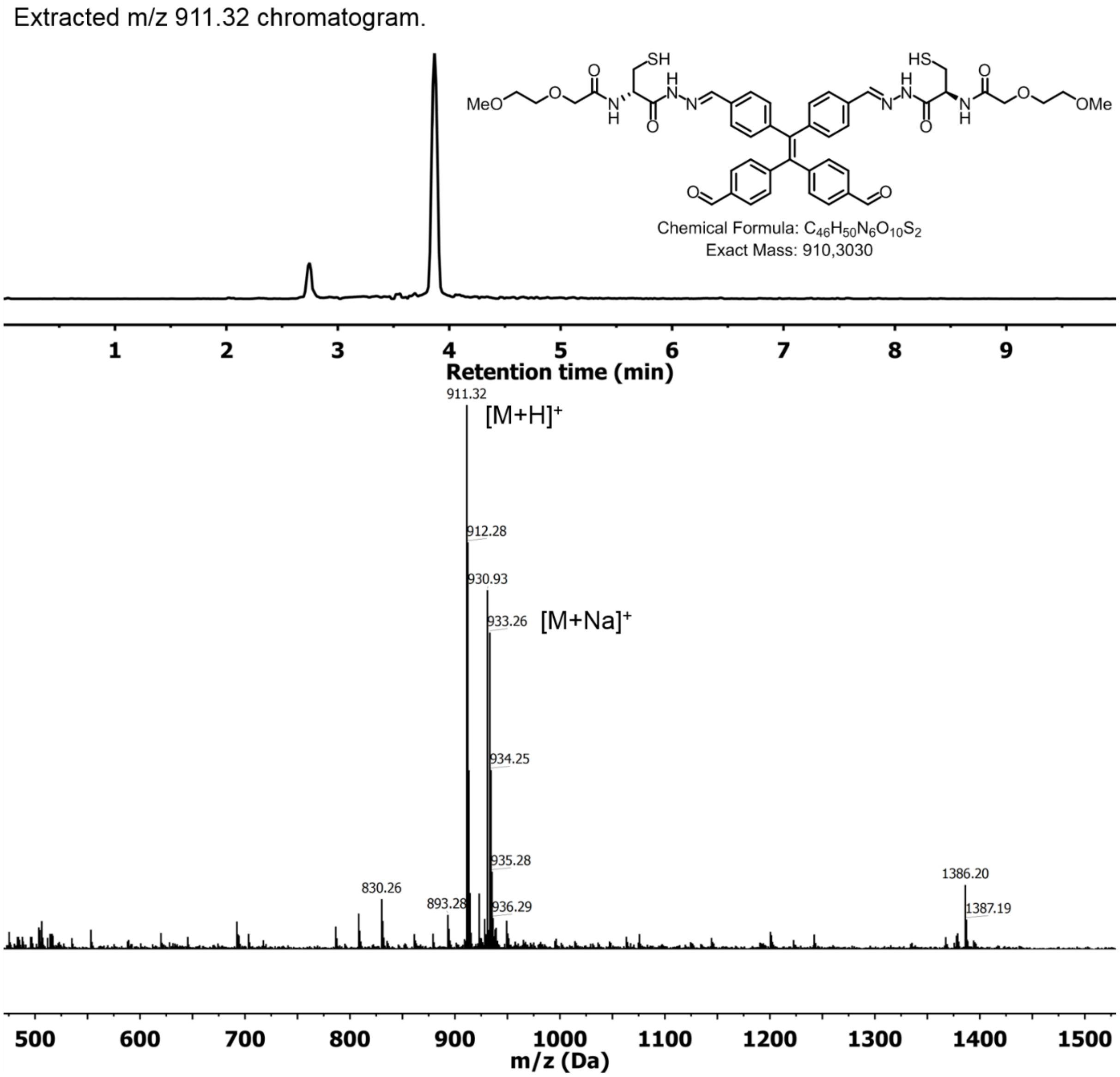

Figure S23. Extracted chromatogram for m/z 911.32 from LC-MS trace of equilibrating mixture of DEG-Cage formation after 24h (top). Mass spectra of DEG-Cage-intermediate, di-acylhydrazone, calculated m/z for $[M+H]^+$ 911.3103, found 911.32, calculated m/z for $[M+Na]^+$ 933.2922, found 933.26 (down).

Extracted m/z 1144.45 chromatogram.

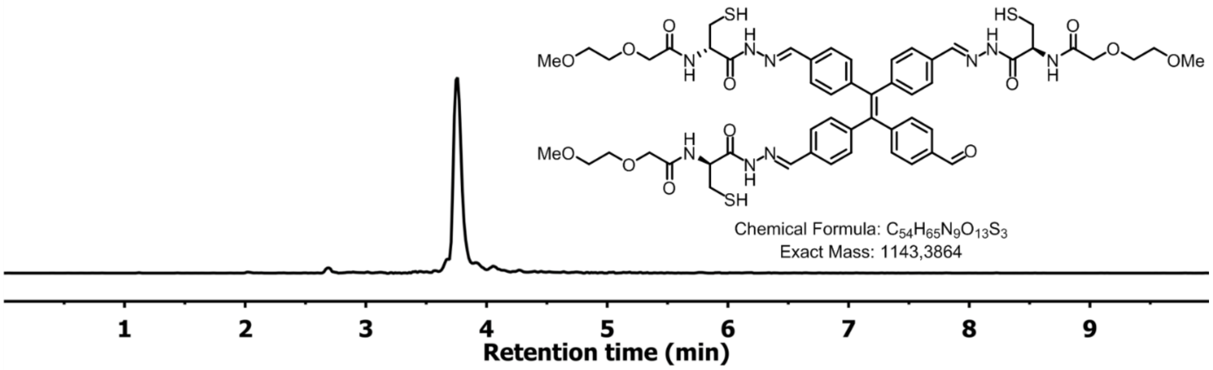

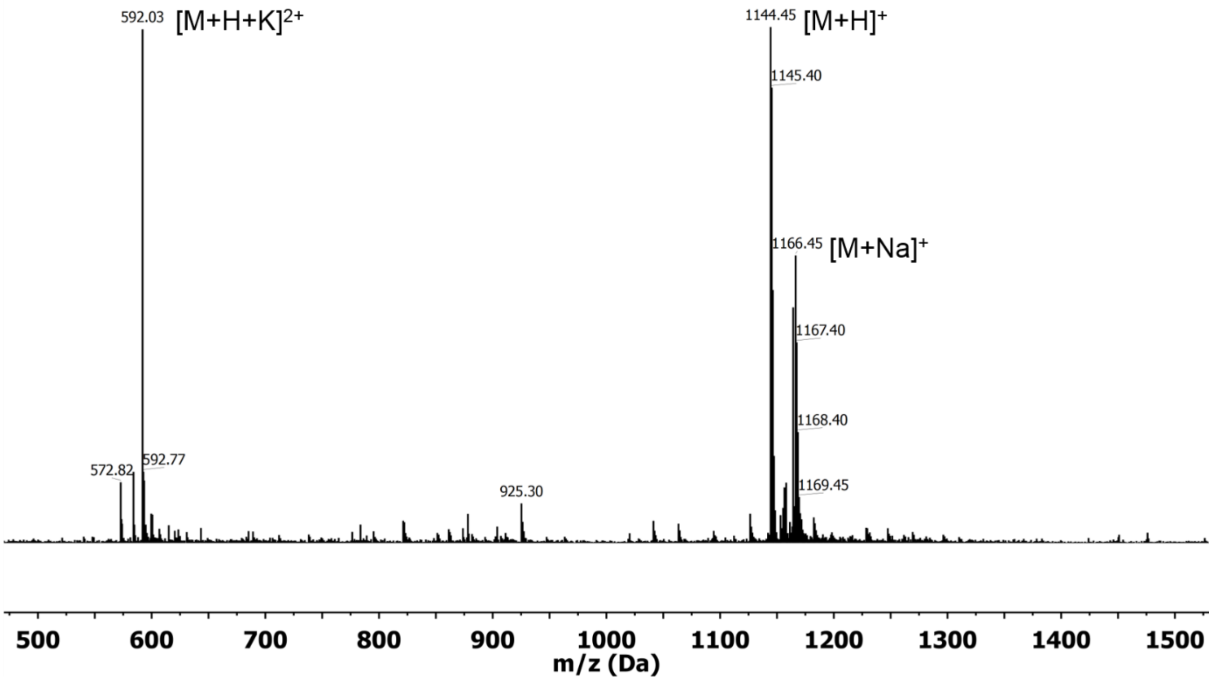

Figure S24. Extracted chromatogram for m/z 1144.45 from LC-MS trace of equilibrating mixture of DEG-Cage formation after 24h (top). Mass spectra of DEG-Cage-intermediate, tri-acylhydrazone, calculated m/z for $[M+H+K]^{2+}$ 591.6784, found 592.03, calculated m/z for $[M+H]^{+}$ 1144.3937, found 1144.45, calculated m/z for $[M+Na]^{+}$ 1166.3756, found 1166.45 (down).

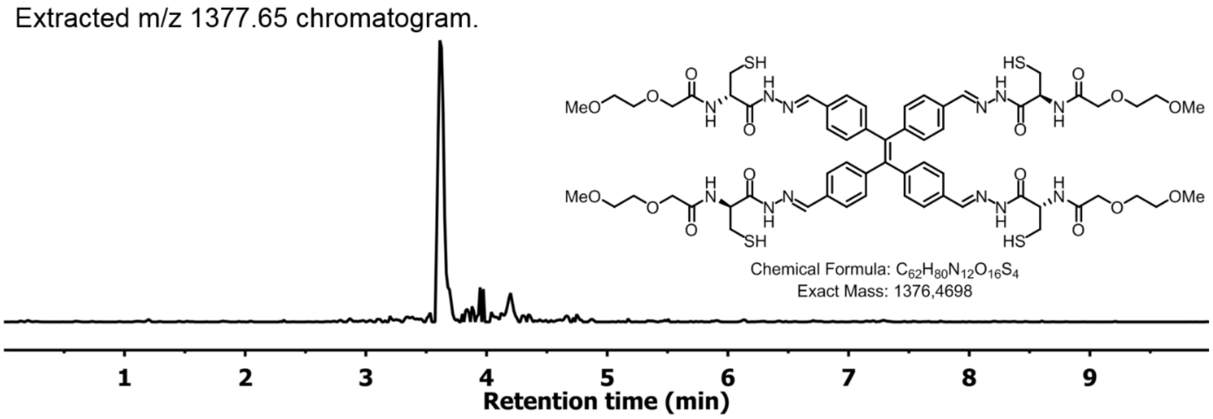

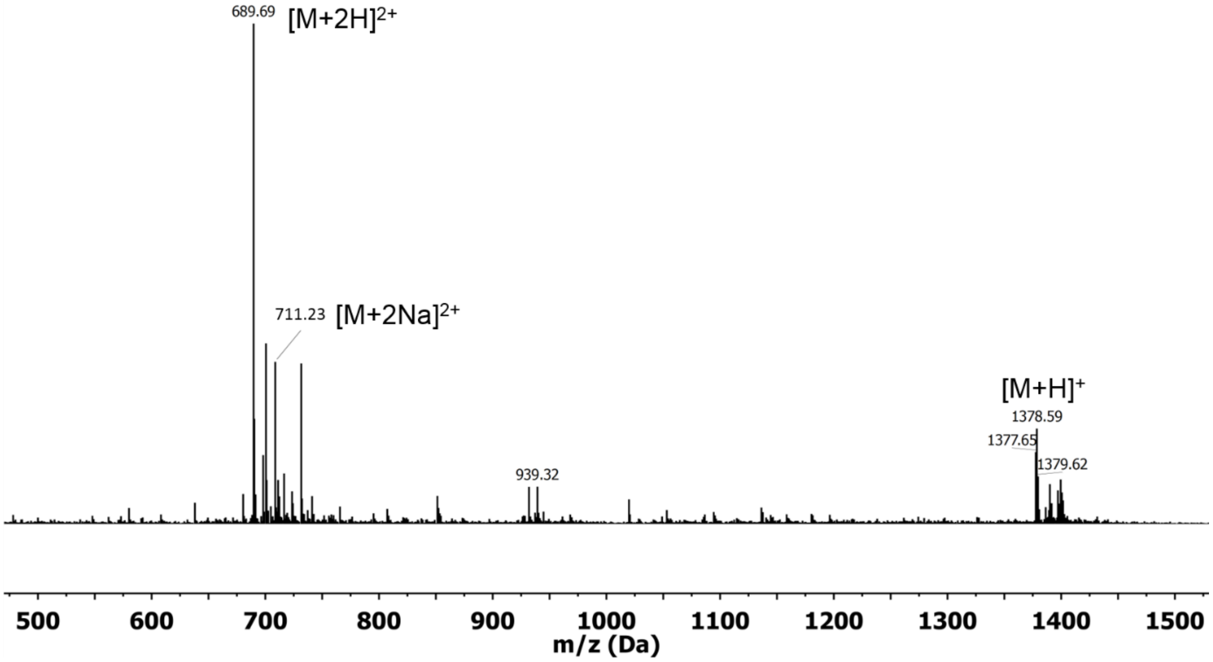

Figure S25. Extracted chromatogram for m/z 1377.65 from LC-MS trace of equilibrating mixture of DEG-Cage formation after 24h (top). Mass spectra of DEG-Cage-intermediate, tetra-acylhydrazone, calculated m/z for $[M+2H]^{2+}$ 689.2422, found 689.69, calculated m/z for $[M+2Na]^{2+}$ 711.2241, found 711.23, calculated m/z for $[M+H]^{+}$ 1377.4771, found 1377.65 (down).

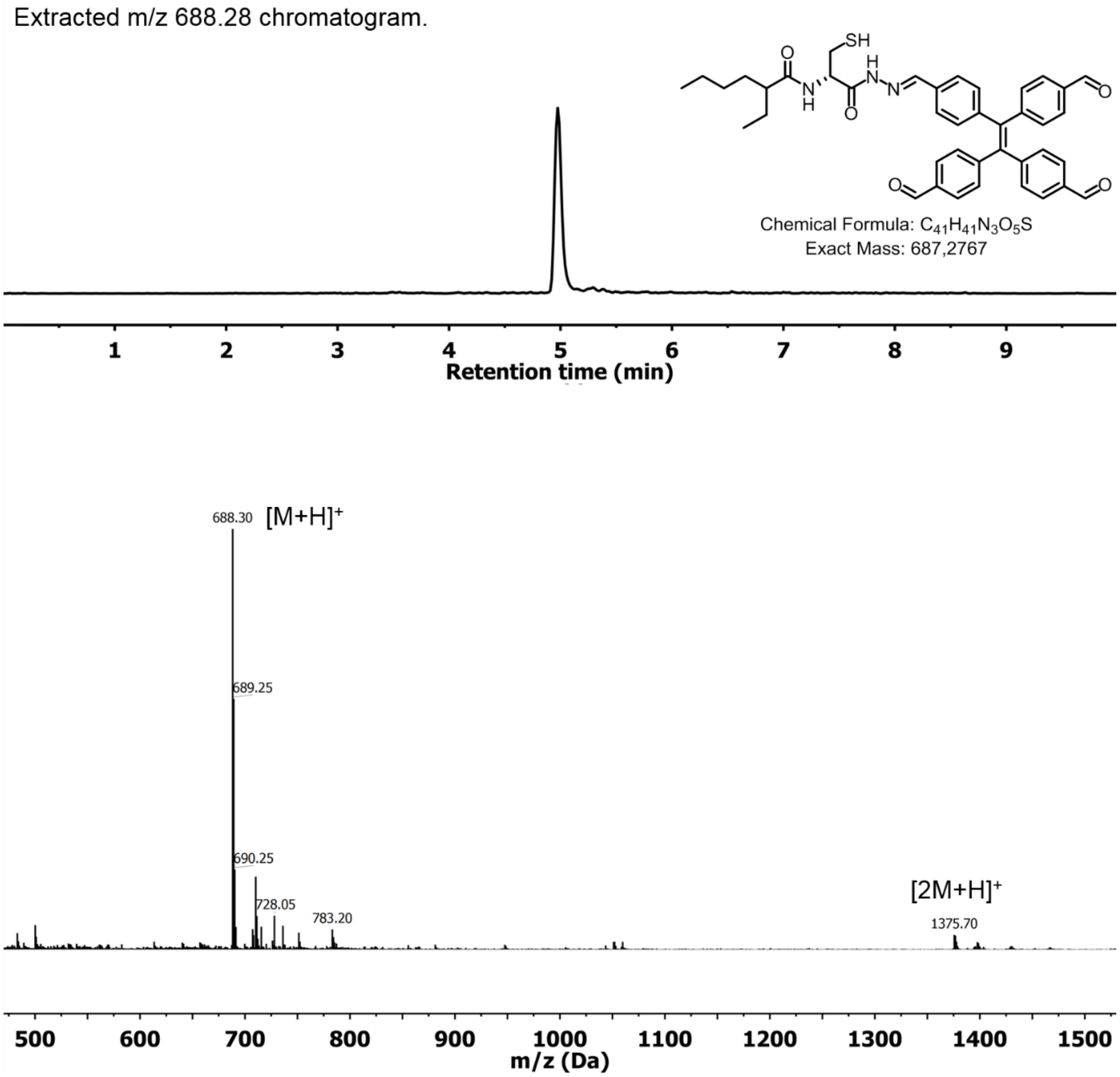

Figure S26. Extracted chromatogram for m/z 688.28 from LC-MS trace of equilibrating mixture **of EH-Cage** formation after 24h (top). Mass spectra of **EH-Cage**-intermediate, mono-acylhydrazone, calculated m/z for $[M+H]^+$ 688.2839, found 688.30, calculated m/z for $[2M+H]^+$ 1375.5607, found 1375.70 (down).

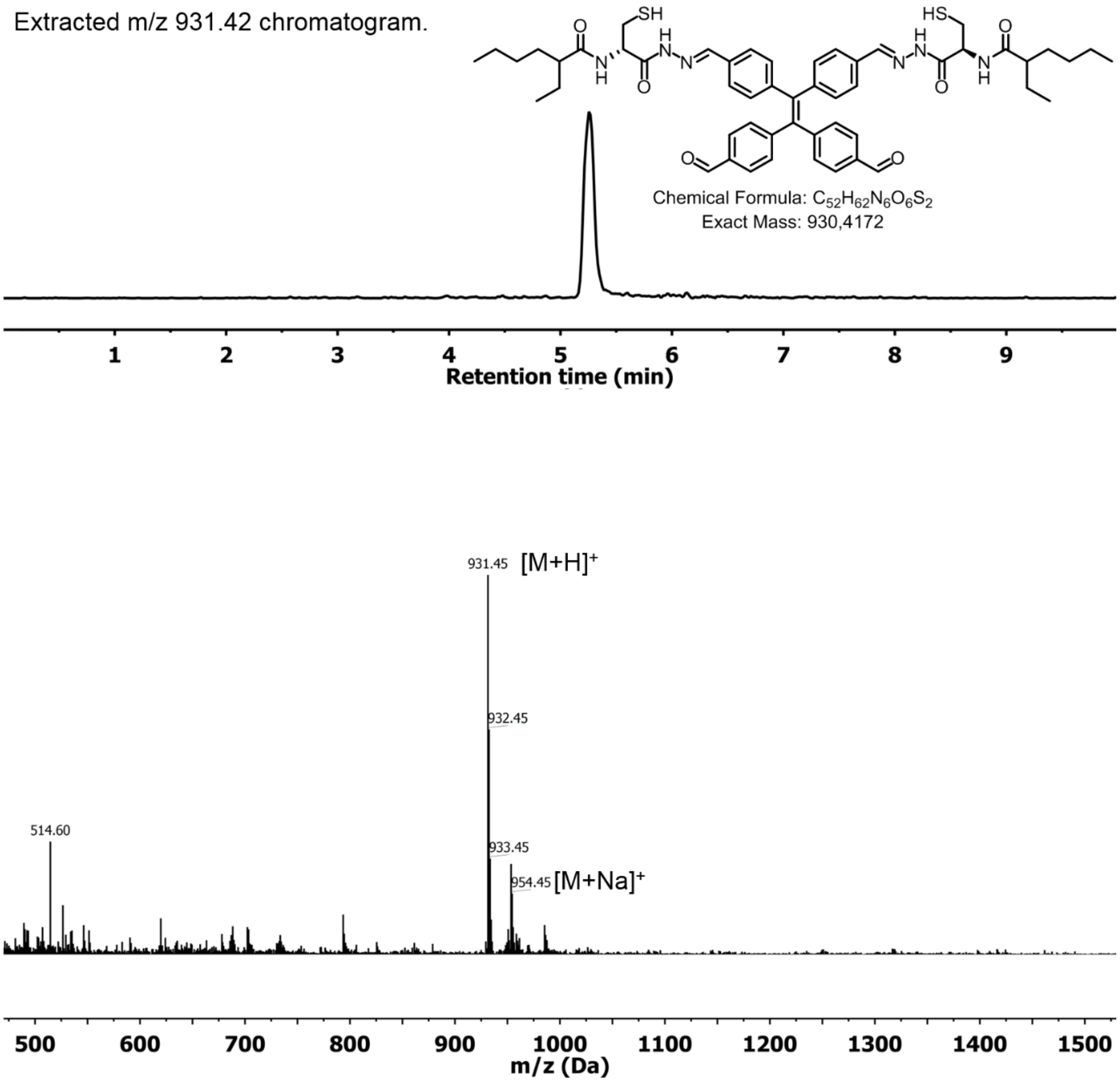

Figure S27. Extracted chromatogram for m/z 931.42 from LC-MS trace of equilibrating mixture **of EH-Cage** formation after 24h (top). Mass spectra of **EH-Cage**-intermediate, di-acylhydrazone, calculated m/z for $[M+H]^+$ 931.4245, found 931.45, calculated m/z for $[M+Na]^+$ 953.4064, found 953.50 (down).

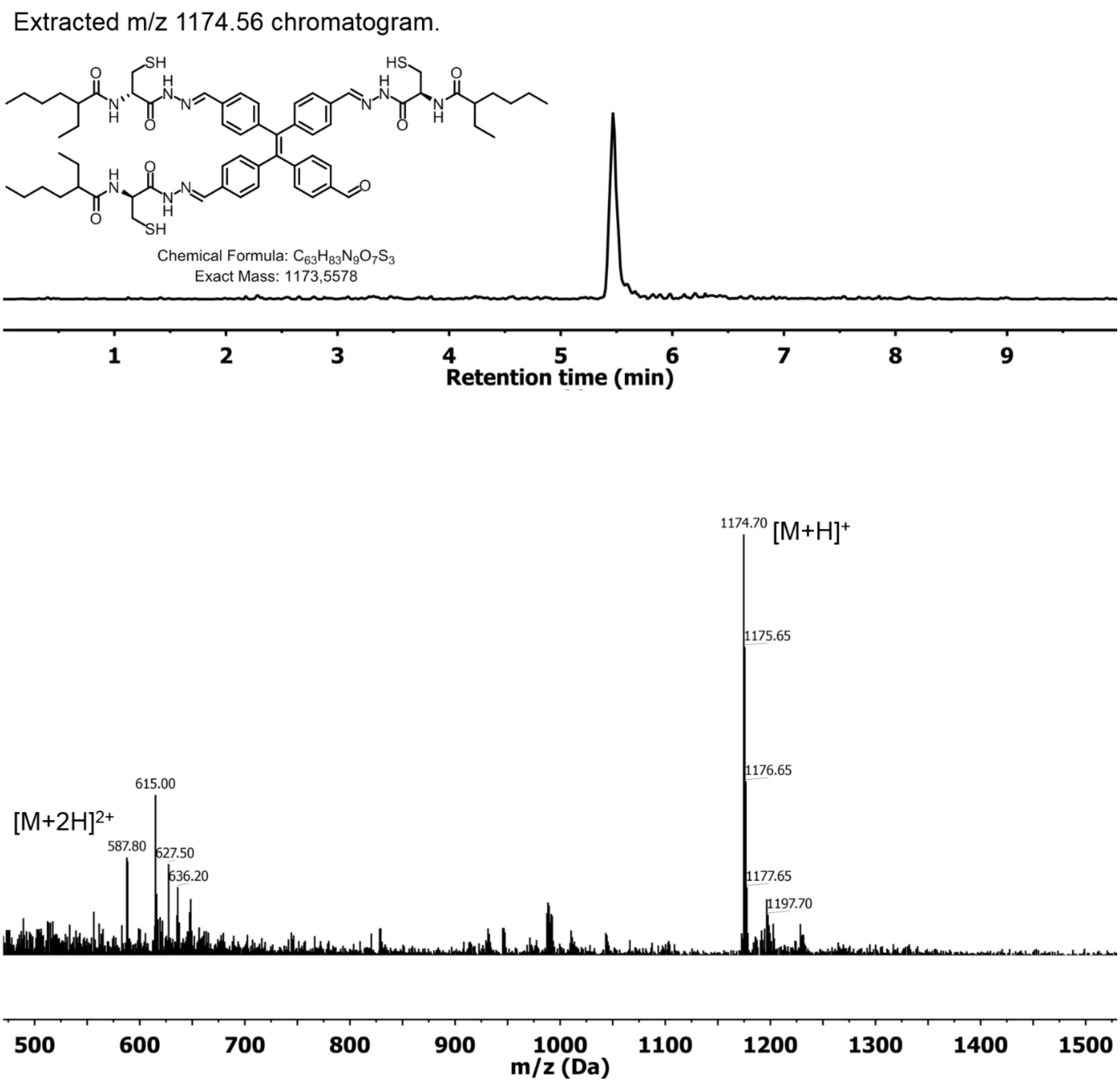

Figure S28. Extracted chromatogram for m/z 1174.56 from LC-MS trace of equilibrating mixture **of EH-Cage** formation after 24h (top). Mass spectra of **EH-Cage**-intermediate, tri-acylhydrazone, calculated m/z for $[M+2H2]^+$ 587.7862, found 587.80, calculated m/z for $[M+H]^+$ 1174.5651, found 1174.70 (down).

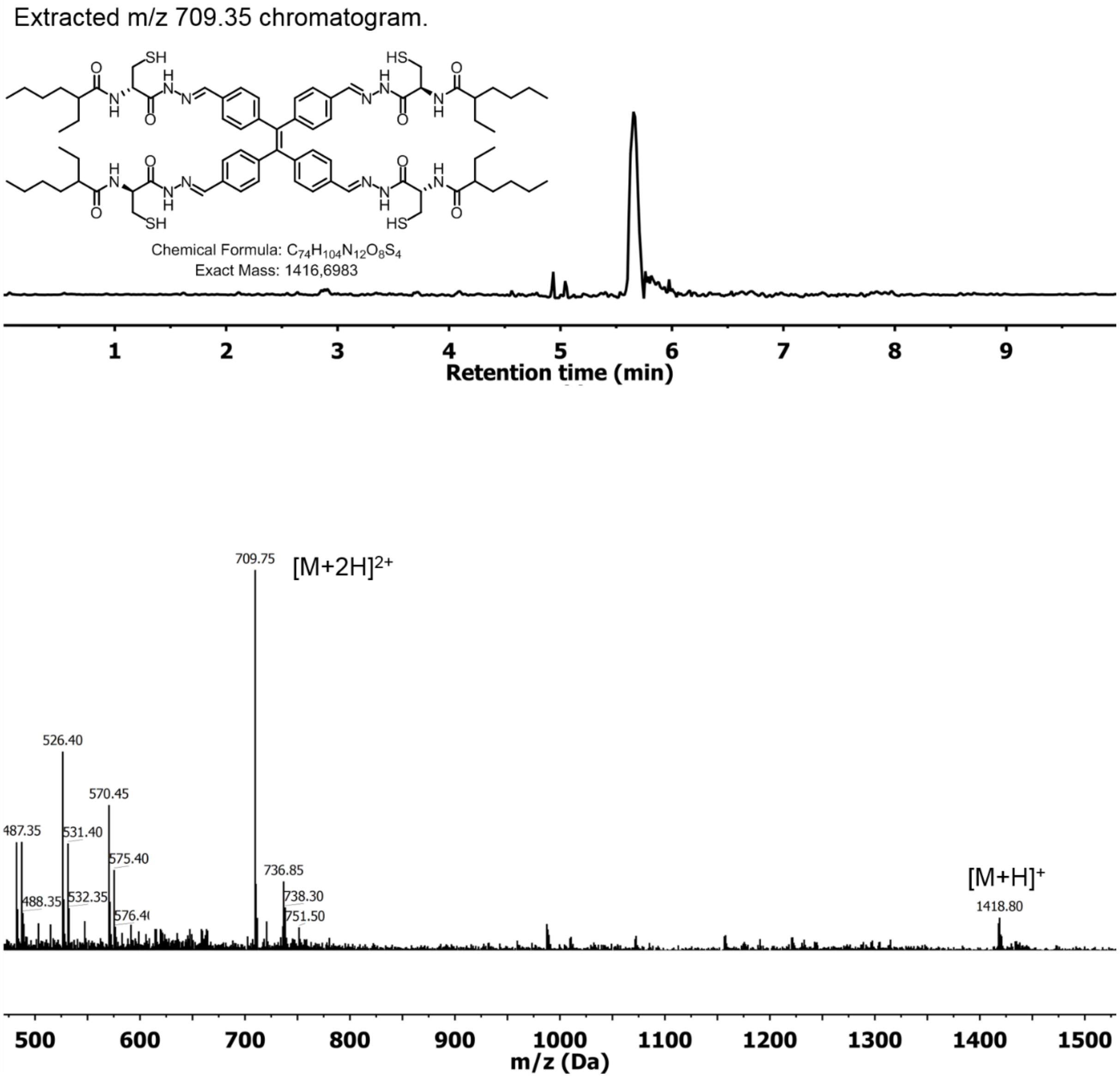

Figure S29. Extracted chromatogram for m/z 709.35 from LC-MS trace of equilibrating mixture **of EH-Cage** formation after 24h (top). Mass spectra of **EH-Cage**-intermediate, tetra-acylhydrazone, calculated m/z for $[M+2H]^{2+}$ 709.3564, found 709.75, calculated m/z for $[M+H]^{+}$ 1417.7056, found 1418.00 (down).

5. NMR Spectra of cages

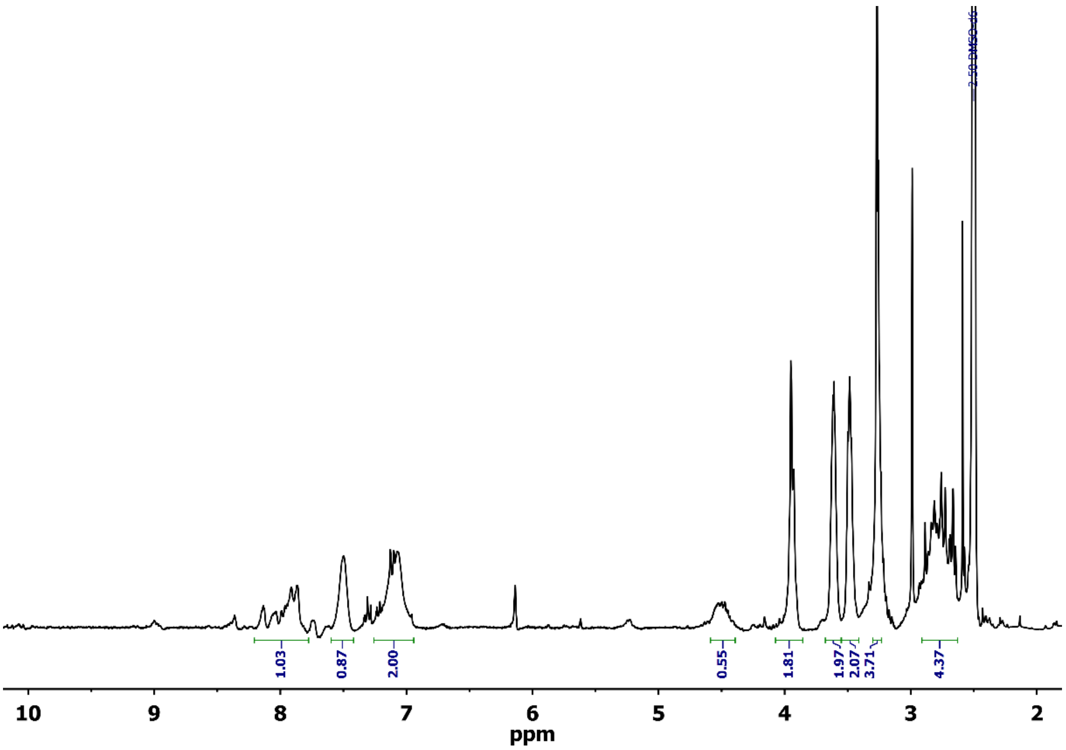

Figure S30. 1H NMR spectra of **DEG-Cage** in DMSO-d_6 at 298K (400 MHz).

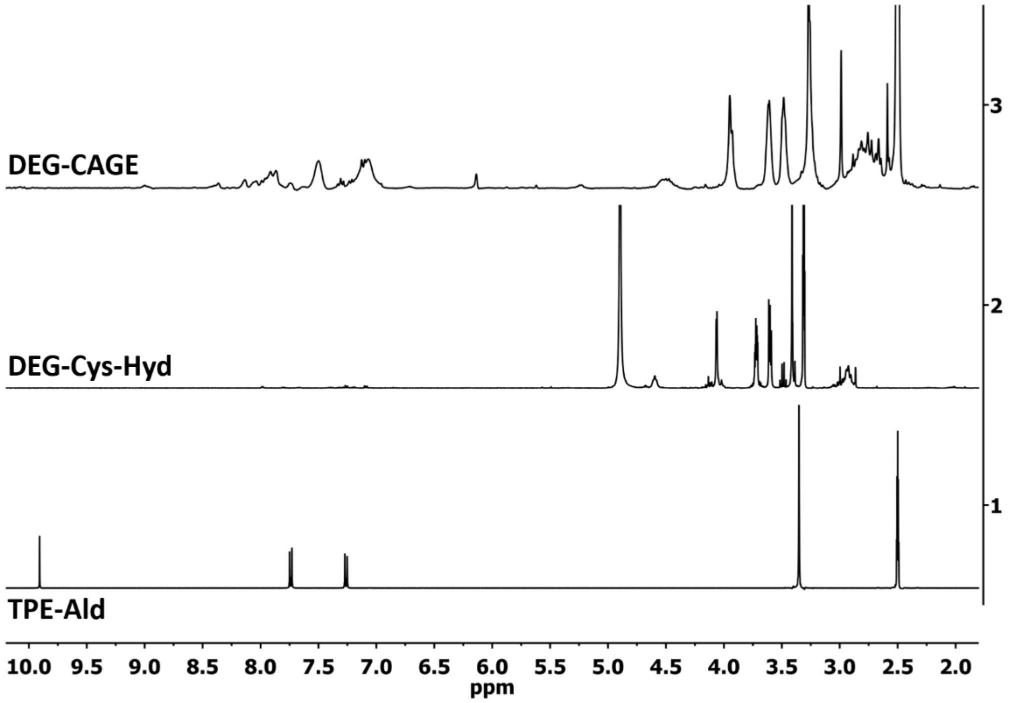

Figure S31. Comparison of 1H NMR spectra of **DEG-Cage** and 1H NMR of substrates **DEG-Cys-Hyd** and **TPE-Ald** at 298K (400 MHz).

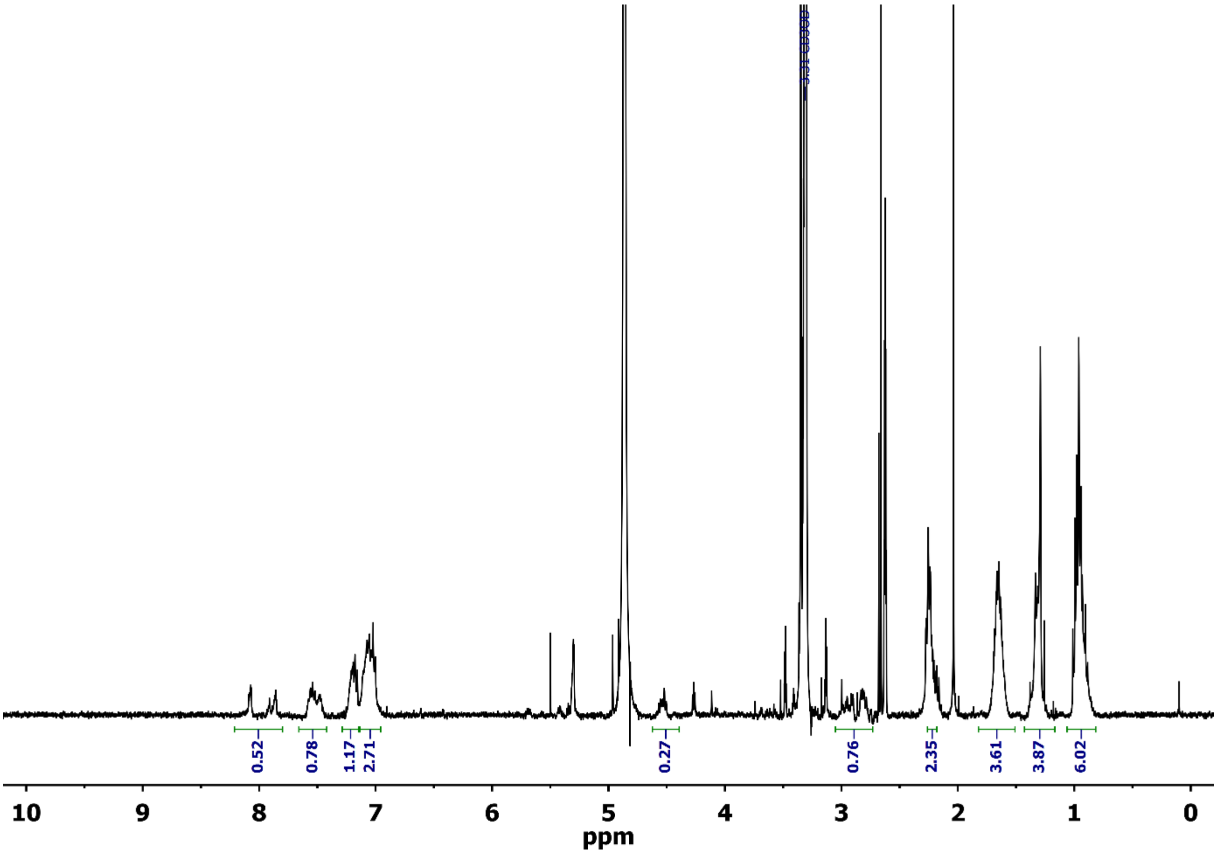

Figure S32. 1H NMR spectra of **EH-Cage** in MeOD-d_4 at 298K (400 MHz).

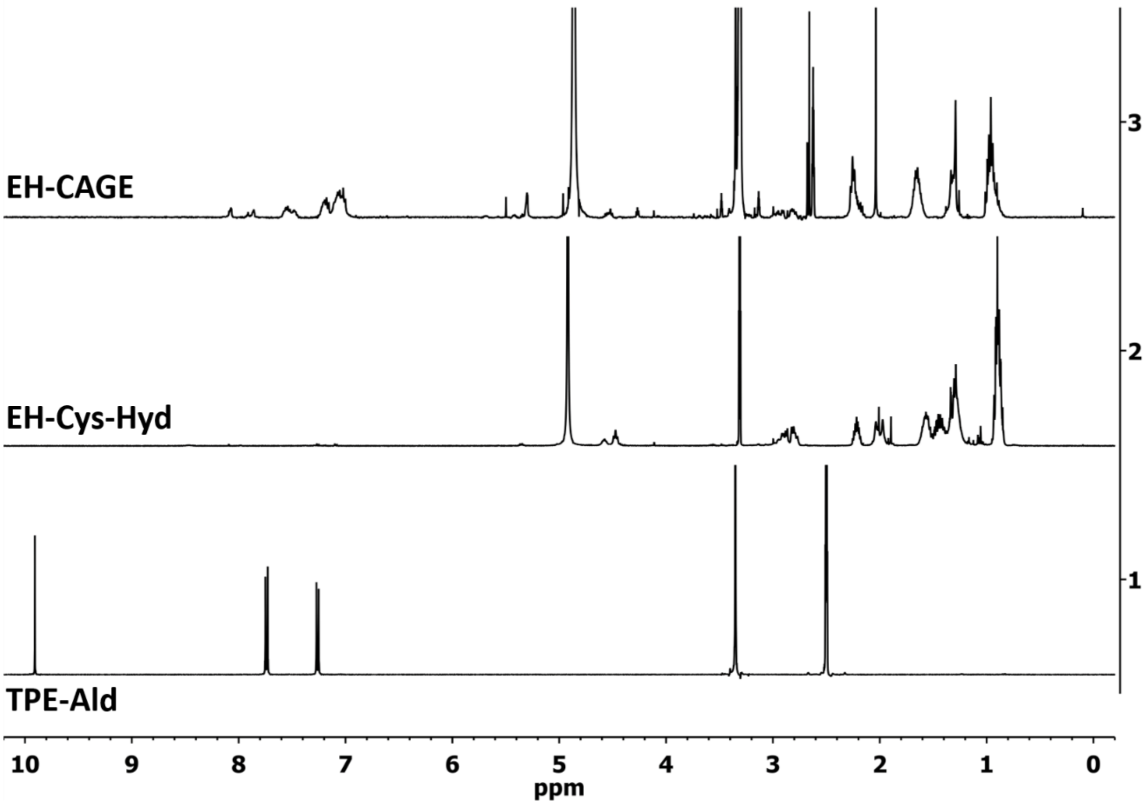

Figure S33. Comparison of 1H NMR spectra of **EH-Cage** and 1H NMR of substrates **EH-Cys-Hyd** and **TPE-Ald** at 298K (400 MHz).

6. Molecular modelling

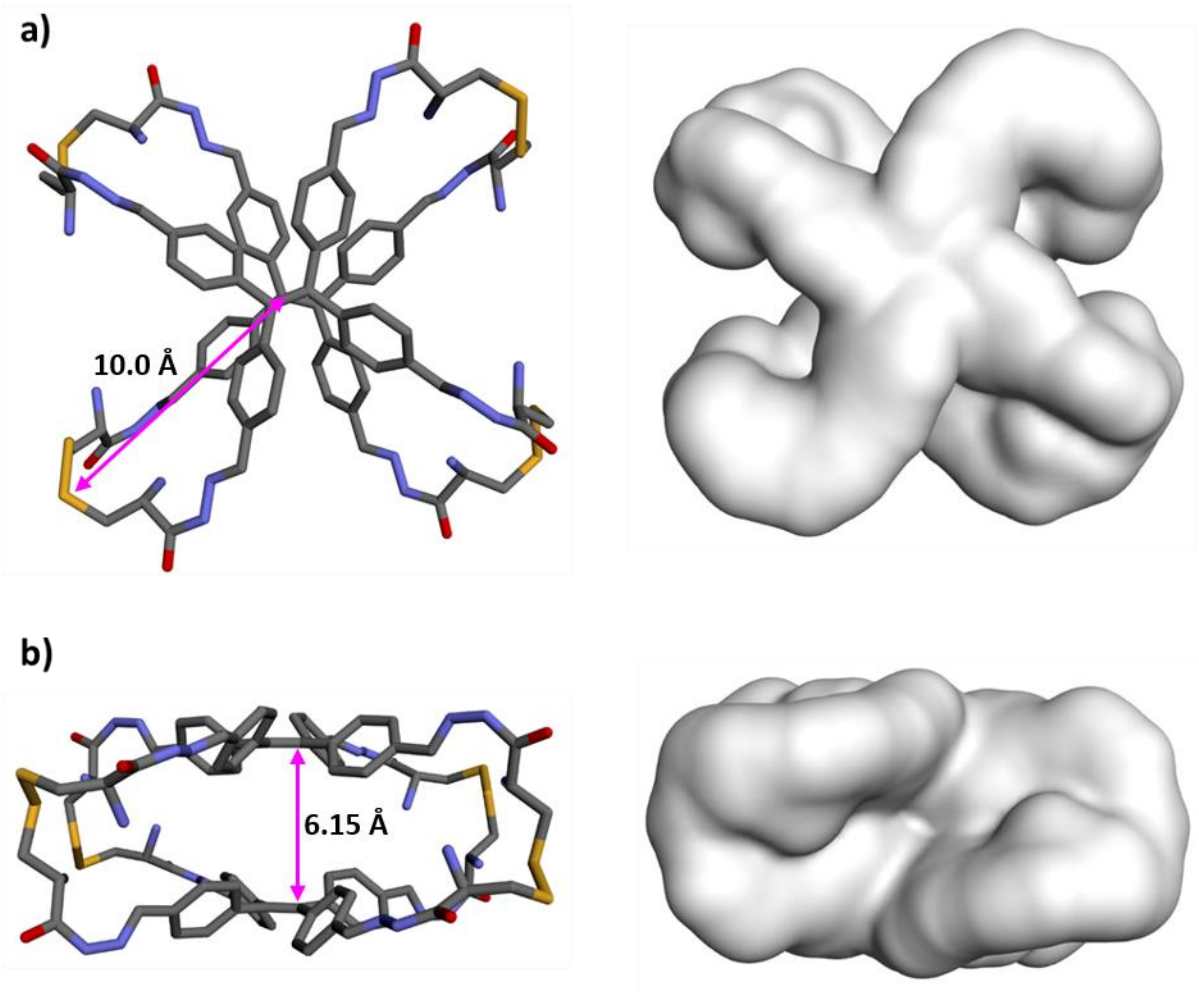

Figure S34. The structure of *N*-unmodified cage optimized using semiempirical method Recife Model 1 (RM1), a reparameterization of AM1 semiempirical method for H, C, N, O, P, S, F, Cl, Br, and I (Rocha, G. B., Freire, R. O., Simas, A. M. and Stewart, J. J., J. Comput. Chem., (2006) 27: 1101-1111.); a) top view, approx. radius 10 Å and b) side view, approx. height 6.15 Å. Estimated spherical volume 2000 Å^3. The CPK surface shows no internal cavity inside the molecule.
